# Characterization of the G protein-coupled receptor kinase 6 promoter reveals a functional CREB binding site

**DOI:** 10.1371/journal.pone.0247087

**Published:** 2021-02-18

**Authors:** Maike Stegen, Andrea Engler, Crista Ochsenfarth, Iris Manthey, Jürgen Peters, Winfried Siffert, Ulrich H. Frey

**Affiliations:** 1 Department of Anaesthesiology and Intensive Care Medicine, Essen University Hospital and University of Duisburg-Essen, Essen, Germany; 2 Department of Anaesthesiology, Operative Intensive Care Medicine, Pain and Palliative Medicine, Marien Hospital Herne, Ruhr-University Bochum, Bochum, Germany; 3 Institute of Pharmacogenetics, Essen University Hospital and University of Duisburg-Essen, Essen, Germany; Cleveland Clinic, UNITED STATES

## Abstract

**Background:**

G protein-coupled receptor kinase 6 (GRK6) is part of the G protein-coupled receptor kinase family, whose members act as key regulators of seven-transmembrane receptor signalling. GRK6 seems to play a role in regulation of inflammatory processes, but mechanisms of transcriptional regulation of GRK6 expression in inflammatory cell lines have not been characterized. Protein kinase C (PKC) signalling is also involved in inflammatory regulation and an impact of PKC activation on GRK6 protein expression was described previously. Thus, the aim of this study was to 1) characterize the *GRK6* promoter, and 2) investigate a potential influence of PKC on GRK6 expression.

**Methods:**

Five deletion constructs of the *GRK6* promoter were cloned. After transient transfection into a human T cell line, promoter activity was assessed using luciferase reporter gene assays. Putative transcription factor binding sites were identified, mutated, and binding was investigated using electrophoretic mobility shift assays (EMSA). Following stimulation with a PKC activator, GRK6 expression on mRNA and protein levels was assessed by reverse transcriptase qPCR and Western blots.

**Results:**

Investigation of the *GRK6* promoter revealed a putative cAMP responsive element (CRE), whose mutation led to decreased promoter activity (p = 0.0006). Functionality of the CRE binding protein (CREB) binding site was verified in EMSA blots. Stimulation with a PKC activator resulted in decreased *GRK6* promoter activity (p = 0.0027), mRNA (p = 0.04) and protein expression.

**Conclusion:**

We characterized the human *GRK6* promoter and identified promoter activity to be influenced by a CREB binding site. PKC might be one determinant contributing to altered GRK6 expression.

## Introduction

G protein-coupled receptor kinases (GRKs) are a family of seven serine-threonine kinases, allocated to three subfamilies, which interact with specific G protein-coupled receptors (GPCR) as well as with downstream signalling pathways [1]. GRK6 is part of the GRK4 subfamily and is expressed in a broad variety of tissues [2]. It undergoes post-translational palmitoylation (reviewed in [1]) and its expression is detectable in membrane and cytosolic subcellular fractions [3]. GRK6A splice variant is also able to translocate into the nucleus [4]. The *GRK6* gene is located on chromosome 5 (5q35) and has a pseudogene on chromosome 13 [5, 6].

The classical function of GRKs is phosphorylation of GPCRs at their cytosolic tail or third cytoplasmatic loop, which leads to binding of specific arrestin molecules and inhibits conventional interaction with G proteins (reviewed in [7]). Subsequently, recruitment of downstream signalling molecules culminates in internalization and potential downregulation of the receptor [7].

GRK6 is highly expressed in immune cells and an alteration in intracellular GRK6 levels has been specifically reported in relation to inflammation (reviewed in [8]). For instance, blood mononuclear cells of patients suffering from rheumatoid arthritis showed decreased GRK6 expression, while cAMP production was increased in these cells [9]. *GRK6* knockout investigations suggested its role as a regulator under inflammatory conditions. For instance, *GRK6* knockout contributed to post-inflammatory visceral hyperalgesia [10]. Alternatively, *GRK6* overexpression attenuated neuropathic pain after chronic nerve constriction injury in rats through suppression of CXCR2 expression [11].

Thus, these data suggest that GRK6 expression is decreased under inflammatory conditions, and this is evoked by hitherto unknown mechanisms. In concert with this hypothesis, an increase in its expression might have a protective effect regarding development of inflammatory hyperalgesia. Therefore, studies addressing GRK6 expression are not only of interest for cellular biology but also of potential clinical relevance.

CpG islands in the *GRK6* promoter were recently reported to be hypermethylated in hypopharyngeal squamous cell carcinoma as well as in lung adenocarcinoma cells [12, 13]. Moreover, a few specific stimuli leading to an up- or downregulation of GRK6 expression have been identified on a transcriptional level. For example, cell stimulation with phorbol 12-myristate 13-acetate (PMA) decreased GRK6 protein expression in a human promyelocytic cell line [14]. However, in human lymphocyte primary cell cultures, PMA stimulation increased GRK6 expression on mRNA and protein levels [14]. PMA is a potent activator of protein kinase C (PKC). Therefore, a contribution of PKC signalling pathways to regulation of GRK6 expression on a transcriptional level is conceivable. Interestingly, members of the PKC family function as mediators of inflammatory processes [15]. There is evidence to suggest that PKC closely interacts with GRKs. Specifically, direct phosphorylation of GRK5 as a member of the GRK4 subfamily by PKC was shown [16]. In this context, the impact of PKC on GRK6 expression requires further investigation.

The aim of this study, therefore, is to 1) characterize the human *GRK6* promoter and 2) identify major regulatory mechanisms of GRK6 expression on the transcriptional level. The human Jurkat T cell line was used as a model for human immune cells involved in inflammatory processes to investigate the influence of PKC activation on GRK6 expression [17].

## Materials and methods

In this study, we characterized the *GRK6* promoter by using deletion constructs of the *GRK6* 5’-untranslated region (UTR). Promoter activity was assessed using luciferase reporter gene assays in a human T cell line. Putative transcription factor binding sites were investigated *in silico* and mutated via site-directed mutagenesis. Functionality of transcription factor binding sites was assessed by electrophoretic mobility shift assays (EMSAs). A potential contribution of PKC activation on GRK6 expression was investigated by stimulation of a human T cell line using phorbol 12-myristate 13-acetate (PMA), and GRK6 expression on mRNA and protein levels was detected by means of reverse transcriptase real-time quantitative polymerase chain reaction (RT-qPCR) and Western blot investigations.

### Human blood samples

DNA was extracted from blood samples of healthy volunteer donors recruited at the University Hospital of Essen following informed consent. The requirement for ethical review was waived by the institutional review board of the Medical Faculty of the University Duisburg-Essen.

### Cloning of the GRK6 promoter

The human *GRK6* (Entrez gene ID 2870) 5’-UTR was cloned via PCR from DNA using the following primers: *GRK6_Sense* 5’-GGACGCCTTCTCCTATTC-3’ and *GRK6_Antisense* 5’-CTCGGCTCGCAGTGAC-3’ (located at positions -1475 to -1458 and -101 to -117 in relation to translation starting point ATG) (Eurofins Genomics, Ebersberg, Germany). *GRK6* 5’-UTR was cloned into *pGEM T-Easy* vector systems (Promega, Madison, WI, USA). Deletion constructs were created using restriction enzymes (New England Biolabs, Frankfurt am Main, Germany), binding at NsiI, AfeI, AvrII, NcoI and PvuII binding sites, indicated in relation to the translation starting point ATG: -1475/-101, -1143/-101, -892/-101, -396/-101, -310/-101, respectively. Fragments were digested by SacI and filled in by Klenow I (catalogue no. EP0051, Thermo Scientific, Waltham, MA, USA). Plasmid constructs were sequenced (GATC, Konstanz, Germany) and subcloned into the *pGL4*.*10* vector system (Promega, Madison, WI, USA), followed by re-sequencing (GATC, Konstanz, Germany). *GRK6* constructs were transformed in DH5α competent *Escherichia coli* cells (Invitrogen, Karlsbad, CA, USA) and cultured on ampicillin treated media. Thereafter, plasmid DNA was extracted using the Endo Free Plasmid Maxi Kit (Qiagen, Hilden, Germany). DNA amount and purity were verified by means of a TrayCell (Hellma, Müllheim, Germany) and a bio-photometer (Eppendorf, Hamburg, Germany). Samples were stored at -20°C until further use.

### Cell culture

Investigations were carried out in human Jurkat T cell cultures (DSMZ no. ACC282, obtained directedly from the German Collection of Microorganisms and Cell Cultures (DSMZ), Braunschweig, Germany) [17]. Jurkat cells were cultivated in Roosevelt Park Memorial Institute Medium (RPMI, Gibco, Grand Island, NY, USA), supplemented with 10% fetal calf serum (FCS, Biochrom, Berlin, Germany). Cells were maintained in a cell culture incubator at 37°C and 5% CO_2_ and cell culture manipulations were carried out in a microbiological safety cabinet.

### Luciferase reporter gene assays

*pGL4*.*10* and *pGL4*.*74* vector systems (Promega, Madison, WI, USA) were used for luciferase assays. *pGL4*.*10* contains a gene sequence encoding firefly luciferase (*Photinus pyralis*), whose expression depends on the activity of upstream *GRK6* 5’-UTR fragments. *pGL4*.*74* functions as transfection control expressing constitutively renilla luciferase *(Renilla reniformis)*. 150 ng of *pGL4*.*10* plasmids, containing a *GRK6* promoter fragment, and 50 ng of *pGL4*.*74* plasmids per well were co-transfected to Jurkat cells using jetPRIME transfection reagent (Polyplus, Illkirch, France). 65,000 cells were seeded in each well of 96 well-plates in 65 μl of RPMI medium 24 hours prior to transfection. Following 6 hours of incubation, transfection reagents were removed. Luciferase measurements were performed with the Dual-Glo Luciferase Assay System (Promega, Madison, WI, USA) according to the manufacturer´s instructions and conducted in a Tristar LB941 microplate reader (Berthold, Bad Wildbad, Germany) 24 hours after transfection of the cells. Cell viability was verified via microscopy prior to transfection and measurements. Results were analysed by comparison of firefly and renilla luciferase activity.

The impact of cell stimulation on *GRK6* promoter activity was investigated by applying 10 nM phorbol 12-myristate 13-acetate (PMA, Sigma-Aldrich, St. Louis, MO, USA), 100 nM CREB inhibitor 666–15 (Calbiochem, Merck, Darmstadt, Germany), 10 nM pan-PKC inhibitor Gö-6983 or vehicle (dimethyl sulfoxide, DMSO) (both Sigma-Aldrich, St. Louis, MO, USA) after cell transfection. PMA, PKC inhibitor and CREB-inhibitor stimulations were carried out under serum free conditions and serum removal took place 6 hours post transfection. All luminescence measurements were performed 18 hours following to the application of stimulants.

### Site-directed mutagenesis

*GRK6* promoter sequence -396/-310 was analysed using MatInspector Software (Genomatix, München, Germany) [18], resulting in two transcription factor binding sites for the cAMP responsive element binding protein 1 (CREB, Entrez gene ID 1385) and the nuclear factor Y-a (NFYA, Entrez gene ID 4800) at positions -348/-356 and -344/-349. By site-directed mutagenesis (Agilent, Santa Clara, CA, USA), putative transcription factor binding sites were mutated on position –369 AACCAGGAAG AGGTG**TG**GTG ATTGGTCCCG CCCCCGTACT –329 (CREB binding site) and -369 AACCAGGAAG AGGTGACGTG ATTG**T**TCCCG CCCCCGTACT -329 (NFYA binding site). Mutated bases are depicted in bold letters.

### Electrophoretic Mobility Shift Assays (EMSAs)

Two hours after stimulation with 100 nM PMA (Sigma-Aldrich, St. Louis, MO, USA), endogenous nuclear protein fraction was extracted from Jurkat cells using the Cell Lytic NuCLEAR Extraction Kit (Sigma-Aldrich, St. Louis, MO, USA). Nuclear extracts were stored at -80°C until use. 5 μg were used per lane. EMSAs were performed utilizing Odyssey EMSA Buffer Kit (Li-Cor Biosciences, Lincoln, NE, USA) in a composition of 10 mM tris, 50 mM potassium chloride, 1.75 mM dithiolthreitol (DTT), 0.25% tween, 5% glycerol, 0.1 mM EDTA, and 2 mg/ml bovine serum albumin. Buffer ingredients were incubated for 35 minutes at room temperature prior to addition of oligonucleotides, followed by 30 minutes of incubation under light-protected conditions after the addition of oligonucleotides (Eurofins Genomics, Ebersberg, Germany). EMSAs were carried out using double stranded oligonucleotide sequences given as sense strand sequence: *GRK6* promoter sequence containing a putative CREB binding site *CREB GRK6*: 5’-AGGAAGAGGTGACGTGATTGGTCCCGC-3’ (position -365/-338 in relation to translation starting point ATG), *CREB positive control*, derived from CREB1 consensus binding site: 5’-AGAGATTGCCTGACGTCAGAGAGCTAG-3’, and an oligonucleotide containing a mutated CREB1 binding site as *CREB negative control*: 5’-AGAGATTGCCTGTGGTCAGAGAGCTAG-3’. Sequences suspected as putative transcription factor binding sites are underlined, mutated bases are underlined twice. Oligonucleotides were labelled with dye DY682 at their 5’ end. Competition was carried out using similar unlabelled oligonucleotides 200 times in excess. Super shift was conducted using 2 μl rabbit anti-CREB1 p43 polyclonal antibody (antibody registry ID AB_91283, catalogue no. AB3006 Chemicon, Tenecula, CA, USA). Pre-incubation with antibody was carried out for 35 minutes at room temperature prior to the addition of oligonucleotides. Electrophoresis was performed with 5% native polyacrylamide gels. Odyssey Classic Imaging System (Li-Cor Biosciences, Lincoln, NE, USA) was used for detection. Images were cropped using the GNU Image Manipulation Program (Free Software Foundation, Boston, MA, USA).

### Real-time PCR

qPCR was conducted to survey endogenous GRK6 mRNA expression. Following seeding of Jurkat cells in concentrations of 800,000 cells/ml in 12-well dishes, stimulations were carried out under serum free conditions with 100 nM PMA (Sigma Aldrich, St. Louis, MO, USA) or DMSO as vehicle (Sigma Aldrich, St. Louis, MO, USA) for 2, 4, 6, and 8 hours. Cellular RNA was extracted using PEQ Gold Total RNA Kit (peqlab VWR International, Erlangen, Germany) and stored at –80°C until use. A TrayCell (Hellma, Müllheim, Germany) and a bio-photometer (Eppendorf, Hamburg, Germany) were used for RNA quantification. Reverse transcription was performed by means of M-MLV reverse transcriptase (Promega, Madison, WI, USA) with 0.75 μg RNA, using a Biometra T-professional thermocycler (Biometra, Göttingen, Germany). Samples were treated with RNasin ribonuclease inhibitor (Promega, Madison, WI, USA). qPCR of cDNA was conducted on a Step one-plus real-time PCR system (Applied Biosystems, Thermo Scientific, Waltham, MA, USA) utilizing Mesa Green qPCR Master Mix (Eurogentec, Seraing, Belgium). Results were analysed using the comparative 2^-ΔΔCt^ method [19]. Results arising from stimulation with PMA were compared to DMSO vehicle control as ΔCt-value. Reference genes were detected (as previously described [20]), resulting in a combination of hypoxanthine phosphoribosyltransferase 1 (HPRT, Entrez Gene ID: 3251) and β-2-microglobulin (B2M, Entrez Gene ID: 567) as most stably expressed reference genes. The following primers (Eurofins Genomics, Ebersberg, Germany) were used:

*Hs_GRK6_forward*: 5’-TTCAAGCGGCTGGGAGC-3’,

*Hs_GRK6_reverse*: 5’-GCCTGTGGCAAACTTCTGGT-3’,

*Hs_HPRT_forward*: 5’-CACTGGCAAAACAATGCAGACT-3’,

*Hs_HPRT_reverse*: 5’-GTCTGGCTTATATCCAACACTTCGT-3’,

*Hs_B2M_forward*: 5’-GCTGGCGGGCATTCCT-3’,

*Hs_B2M_reverse*: 5’-AATCTTTGGAGTACGCTGGATAGC-3’.

*mRNA stability* was investigated using 5 μg/ml actinomycin D (Calbiochem, Merck Millipore, Darmstadt, Germany) and 20 μg/ml cycloheximide (Sigma-Aldrich, St. Louis, MO, USA). Stimulations were carried out in serum starved Jurkat cells for 6 hours. RNA extraction and reverse transcription were handled as mentioned before.

### Western blot

Jurkat cells were seeded in 10 cm cell culture dishes at a concentration of 1x10^6^ cells/ml. Following stimulation with 100 nM PMA or vehicle (DMSO) (Sigma-Aldrich, St. Louis, MO, USA) for 2, 4, or 6 hours, and following stimulation with 100 nM CREB inhibitor 666–15, actinomycin D (5 μg/ml) (both Calbiochem, Merck, Darmstadt, Germany) and cycloheximide (20 μg/ml, Sigma-Aldrich, St. Louis, MO, USA) for 6 hours respectively, total protein extraction was carried out using RIPA lysis buffer (150 mM sodium chloride, 1% NP40, 0.5% sodium deoxycholate, 0.1% SDS, 50 mM Tris pH 8.0) and complete mini protease inhibitor cocktail (Roche Diagnostics, Mannheim, Germany). Cells were washed twice using pre-chilled Dulbecco’s phosphate buffered saline (DPBS, Gibco, Grand Island, NY, USA), the cell pellet was resuspended in 300 μl of RIPA lysis buffer plus protease inhibitor cocktail and spun briefly for 10 seconds, followed by incubation on ice for 30 minutes. After centrifugation for 20 minutes at 12,000g and 4°C, supernatants were stored at -80°C until use. The amount of extracted protein was determined using the Pierce BCA Protein Assay Kit (Thermo Scientific, Rockford, IL, USA). Electrophoresis was carried out on 10% sodium dodecyl-sulphate polyacrylamide gels under application of 40 μg of protein per lane. Membranes were incubated with either rabbit anti-GRK6 monoclonal antibody D1A4 (antibody registry ID AB_11179210, catalogue no. 5878S, Cell Signaling, Danvers, MA, USA), final dilution of 1:1,000 in TBS-T, supplemented with 5% dried milk powder, or–following membrane stripping–mouse anti-actin β monoclonal antibody (antibody registry ID AB_626632, catalogue no. sc-47778, Santa Cruz, Dallas, TX, USA), final dilution 1:1,000 in TBS-T plus 5% dried milk powder. Used as secondary antibodies were goat anti-mouse IgG (horseradish peroxidase conjugated) polyclonal antibody (antibody registry ID AB_631736, catalogue no. sc-2005, Santa Cruz, Dallas, TX, USA), dilution 1:10,000 in TBS-T containing 5% dried milk powder, and goat anti-rabbit IgG (horseradish peroxidase conjugated) polyclonal antibody (antibody registry ID AB_631746, catalogue no. sc-2004, Santa Cruz, Dallas, TX, USA), dilution 1:5,000 in TBS-T cont aining 5% dried milk powder. Signal detection was carried out either on radiographic films or by use of the ChemiDoc XRS+ Gel Imaging System (Biorad, Hercules, CA, USA) and Signal Fire TM Plus Reagent (Cell Signaling, Danvers, MA, USA). Images were cropped and gel quantification was carried out by using the Image Lab 6.1 Software (Biorad, Hercules, CA, USA).

### Statistics

All measurements were carried out in duplicates to improve reliability of measurements, results averaged, and depicted as means ± standard error of the mean. Wilcoxon matched pairs ranked sign tests, Mann-Whitney-U-test, and one-way or two-way ANOVA with Sidak’s or Bonferroni’s multiple comparisons post hoc tests were applied for statistical analysis, as appropriate. Predetermined alpha-errors p of less than 0.05 were considered statistically significant. Data analysis was performed using Graph Pad Prism software version 7.04 (La Jolla, CA, USA).

## Results

### Identification of the GRK6 promoter and determination of a functional CREB binding site

**Fig 1:**
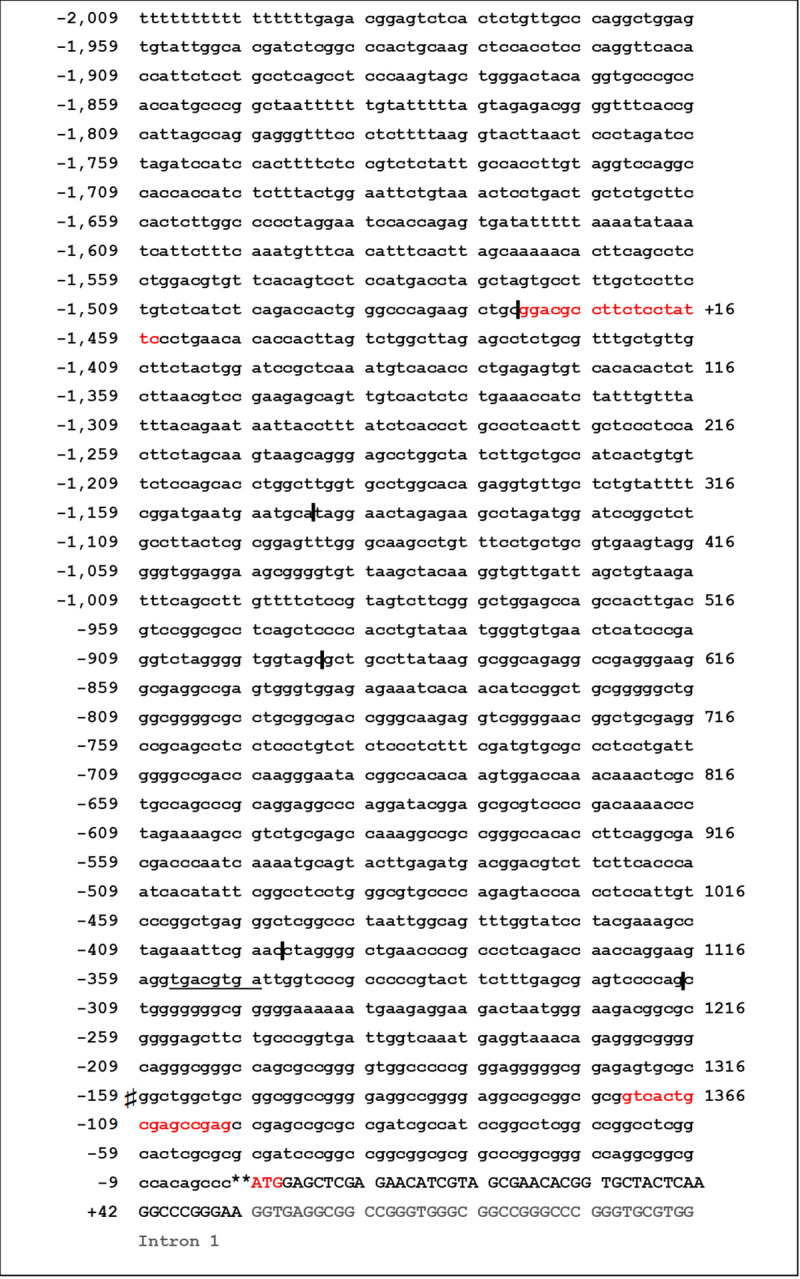
Schematic overview of *GRK6* 5’-UTR sequence. The following symbols were used to describe the below-mentioned terms: ♯ Transcription starting point. ** Translation starting point at sequence triplet ATG (marked red). I Sites of restriction enzyme intersection, used to create deletion constructs. All negative signed inscriptions are designated in relation to the translation starting point. Red letters in lower case mark primer binding sites in the promoter for initial cloning. Capital letters are used from translation starting point onwards. Light grey letters display intron sequence 1. The underlined sequence shows a putative CREB binding site.

**Fig 2:**
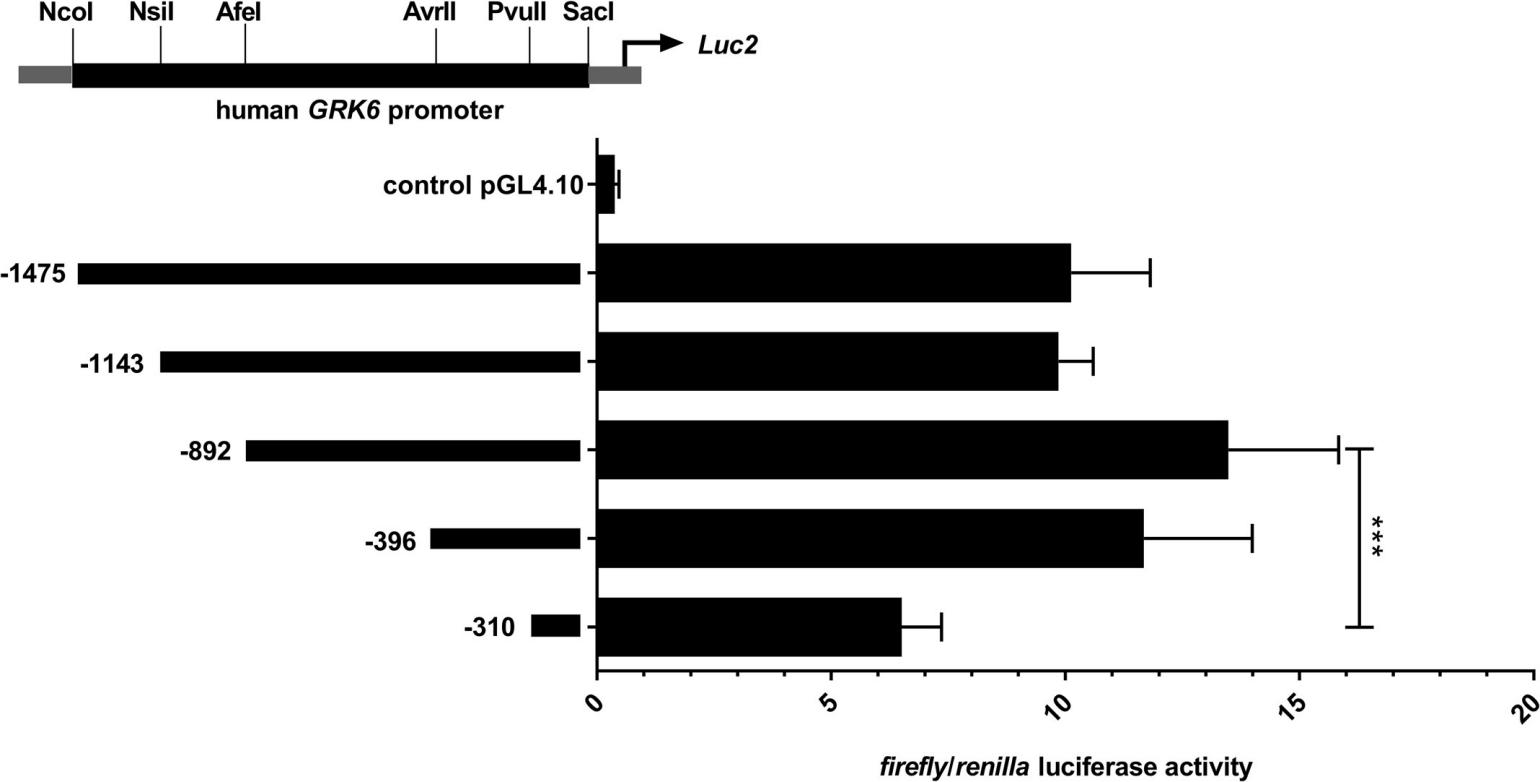
Comparative activity of five deletion constructs of the *GRK6* promoter. Construct -396 (in relation to ATG as translational starting point) showed the greatest increase in promoter activity compared to the next shorter construct. N = 7, measured in duplicate. Friedman test with Dunn’s multiple comparison test. ***p = 0.0003.

**Fig 3:**
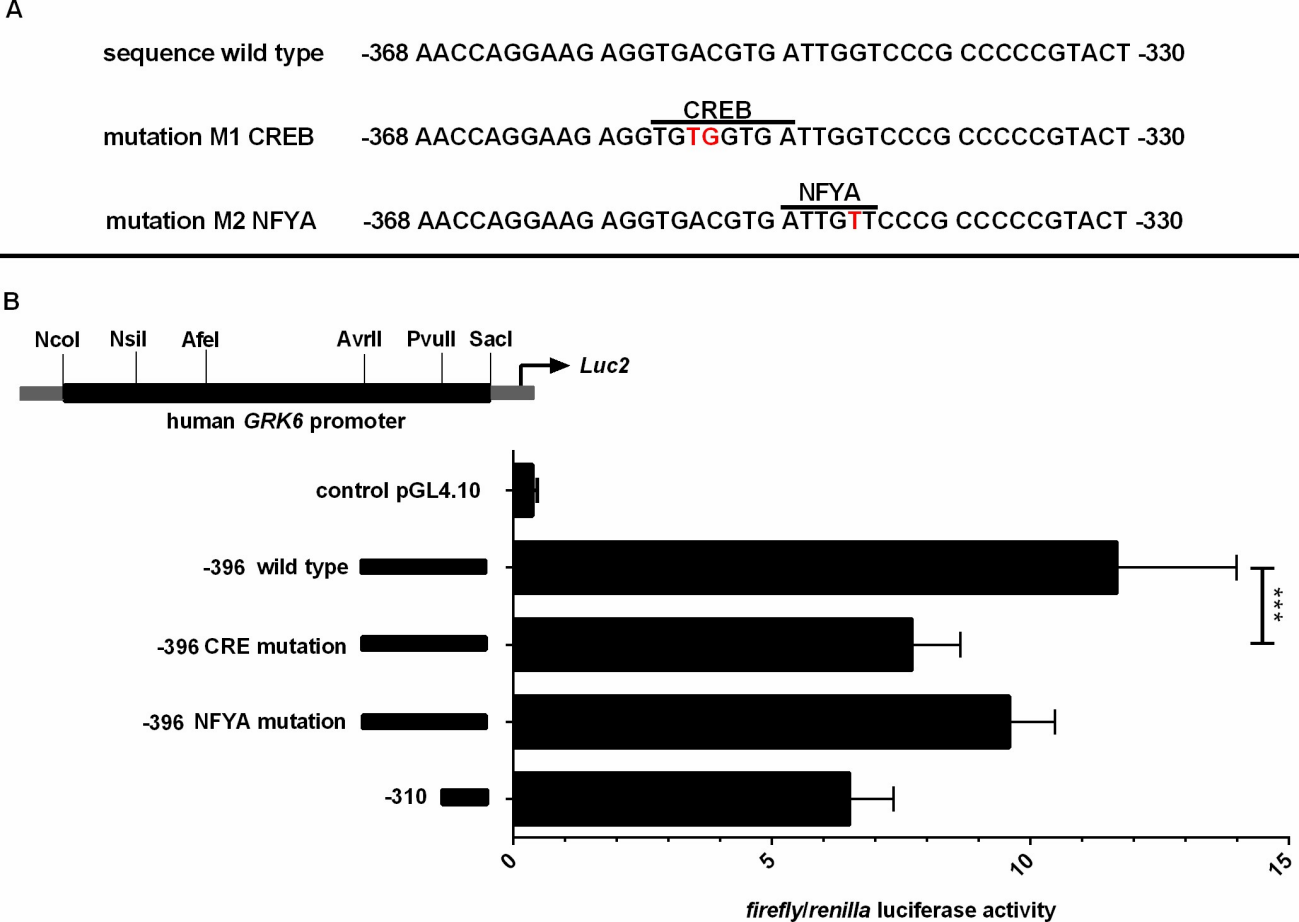
(A) Scheme of mutations of putative transcription factor binding sites in *GRK6* promoter. Inscriptions in relation to the translation starting point ATG. Mutated bases are marked in bold red letters. (B) Site directed mutagenesis of CREB binding site yielded significant downregulation of *GRK6* promoter activity. Mutation of putative CREB resp. NFYA binding sites in construct -396 leads to a decrease in promoter activity in comparison to wild type promoter sequences, especially following mutation of the CRE sequence. N = 7, measured in duplicate. Wilcoxon matched pairs signed rank test. ***p = 0.0006.

The genomic sequence of the *GRK6* 5’-UTR including relevant nucleotide positions is shown in Fig 1. The transcription start point is located -160 nt upstream from the translational start site ATG [21]. Digestion with diverse restriction enzymes resulted in five different deletion constructs (Fig 2). Measurements of their reporter activity by means of luciferase assays revealed an approximately 80% increase (fold change 1.79) in reporter activity in construct -396 in comparison to the next shorter construct -310, indicated in relation to the translational starting point ATG (Fig 2). This suggested a promoter activity determining region within the section -310 to -396. Indeed, *in silico* analyses of this -310 to -396 sequence using MatInspector software (Genomatix, Munic, Germany) pointed out different putative transcription factor bindings sites, inter alia for CREB and NFYA (Fig 3A). Site directed mutagenesis of both binding sites and determination of their reporter activity via luciferase assays showed a decrease in reporter activity compared to the wild type construct (Fig 3B). Mutation of the CREB binding site yielded a significant decrease in reporter activity (p = 0.0006) suggesting that this CREB binding site might play a pivotal role in *GRK6* promoter regulation. Mutation of the NFYA binding site, however, did not evoke a significant decrease in reporter activity. Nevertheless, a slight but non-significant decrease in activity was detectable, suggesting a minor role of NFYA in the process of *GRK6* promoter activity regulation.

**Fig 4:**
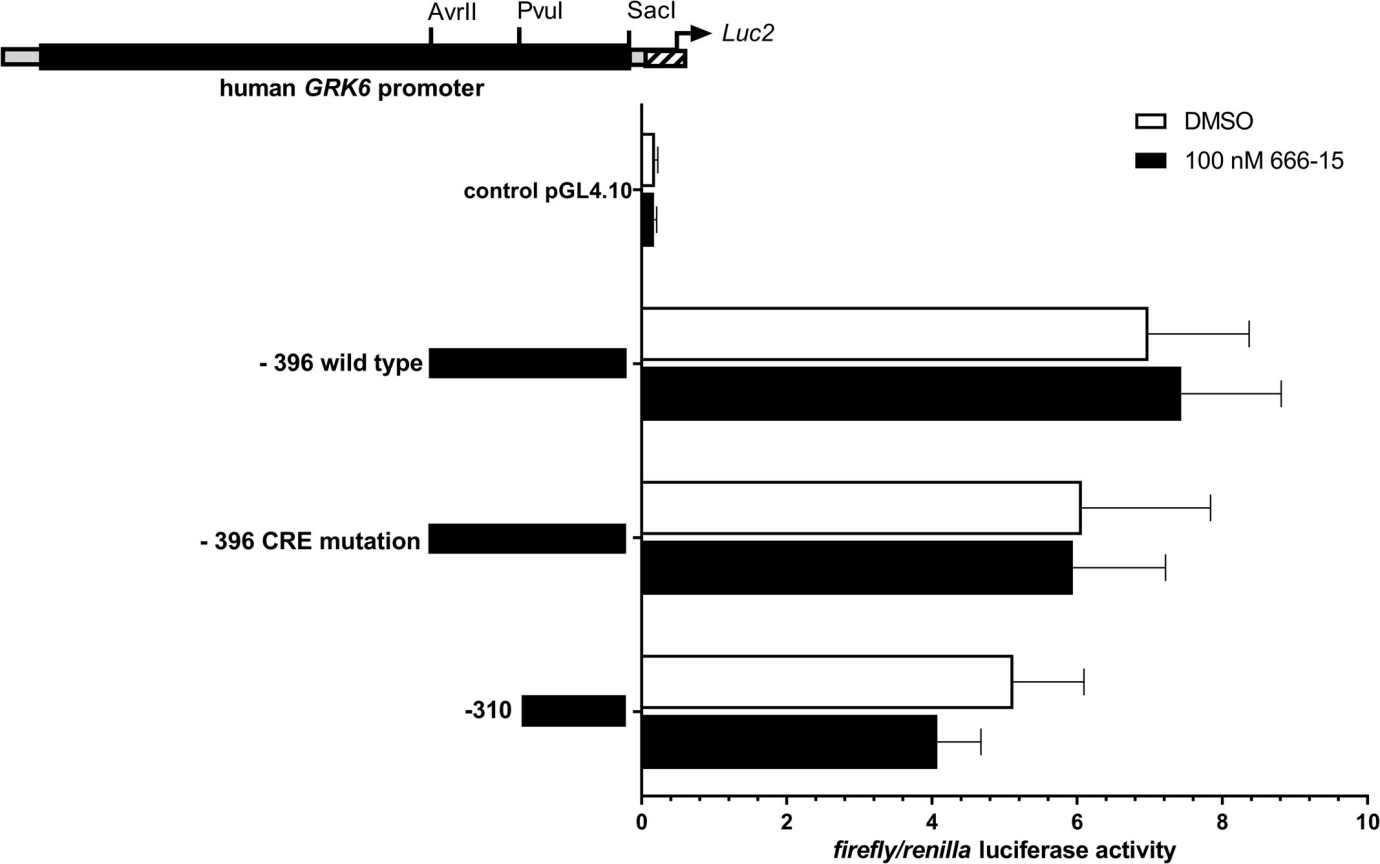
Stimulation with CREB inhibitor 666–15 did not lead to a significant reduction in *GRK6* promoter acitivity in comparison to vehicle control with DMSO. Treatment of serum starved Jurkat cells with CREB inhibitor 666–15 in a concentration of 100 nM. N = 4, measured in duplicates. Depicted are means and standard errors of the mean. Two-way ANOVA and Sidak’s multiple comparison test.

In addition, Jurkat cells were treated with 100 nM of the CREB inhibitor 666–15 and promoter activity was detected by means of luciferase assays (Fig 4) [22]. 666–15 is a potent and specific CREB inhibitor [23]. Notably, treatment with 666–15 did not alter *GRK6* promoter activity in comparison to vehicle control DMSO (Fig 4). Only a slight decrease in *GRK6* promoter activity was detected in construct -310, not containing the putative CREB binding site.

**Fig 5:**
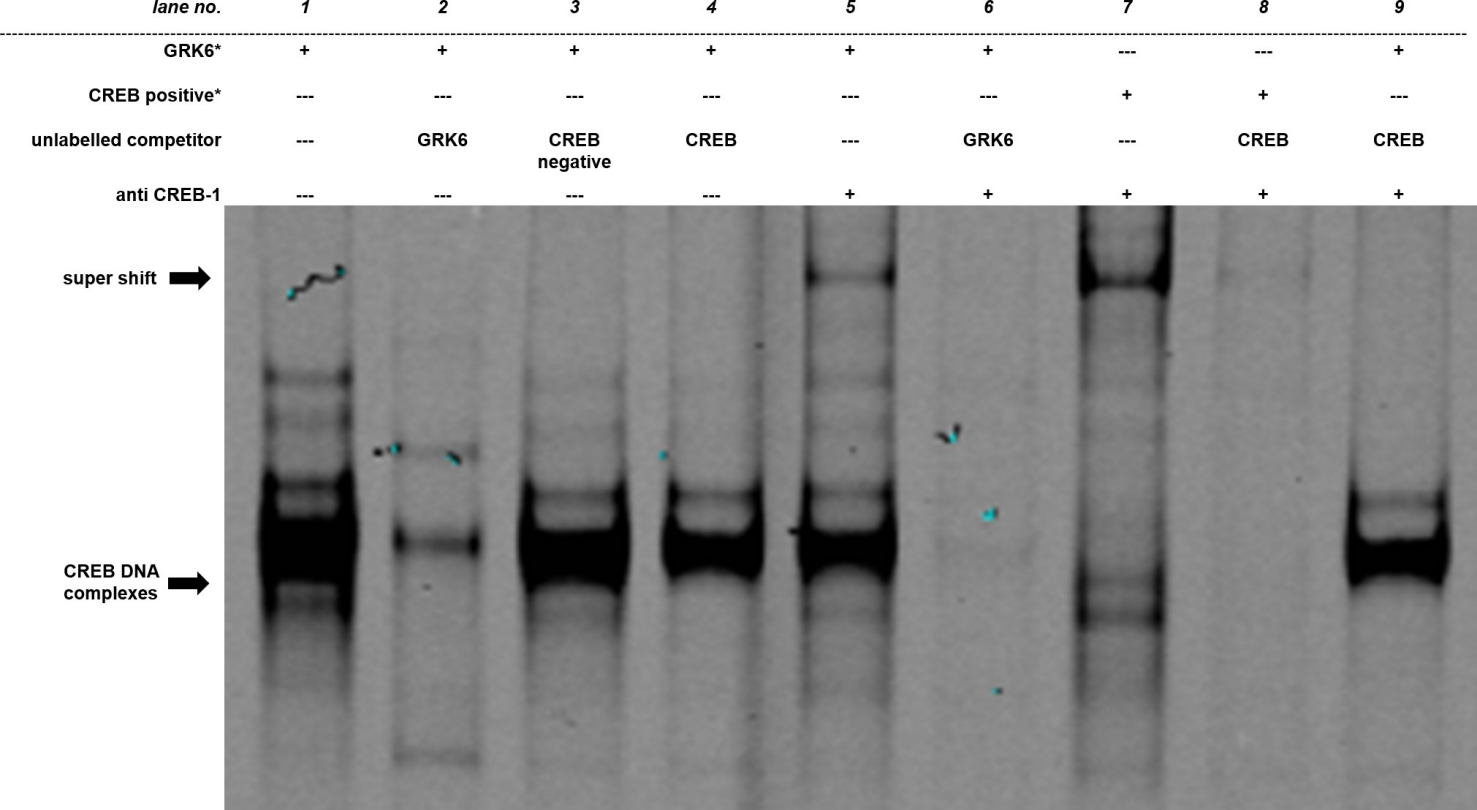
Electrophoretic mobility shift assay outlined specific CREB binding site. Each lane contains 5 μg of nuclear protein extracted from Jurkat cells. Fluorescence labelled GRK6 oligonucleotides (GRK6*, lanes 1–6 and 9) or CREB1 positive controls (CREB positive*, lanes 7 and 8), marked by an asterisk, were used for binding detection. Unlabelled oligonucleotides, either containing the underlying *GRK6* sequence (*GRK6*, lanes 2 and 6), CREB1 positive control (*CREB*, lanes 4, 8, 9) or CREB1 negative control (*CREB negative*, lane 3) without suitable binding sites, were applied in 200 times excess as competitors to unravel potential unspecific binding. Concerning the super shift, 2 μg of anti CREB-1 antibody were added per lane (lanes 5–9) to verify specific binding. N = 3. This image is cropped. The full-length image is shown in S1A Fig.

**Fig 6:**
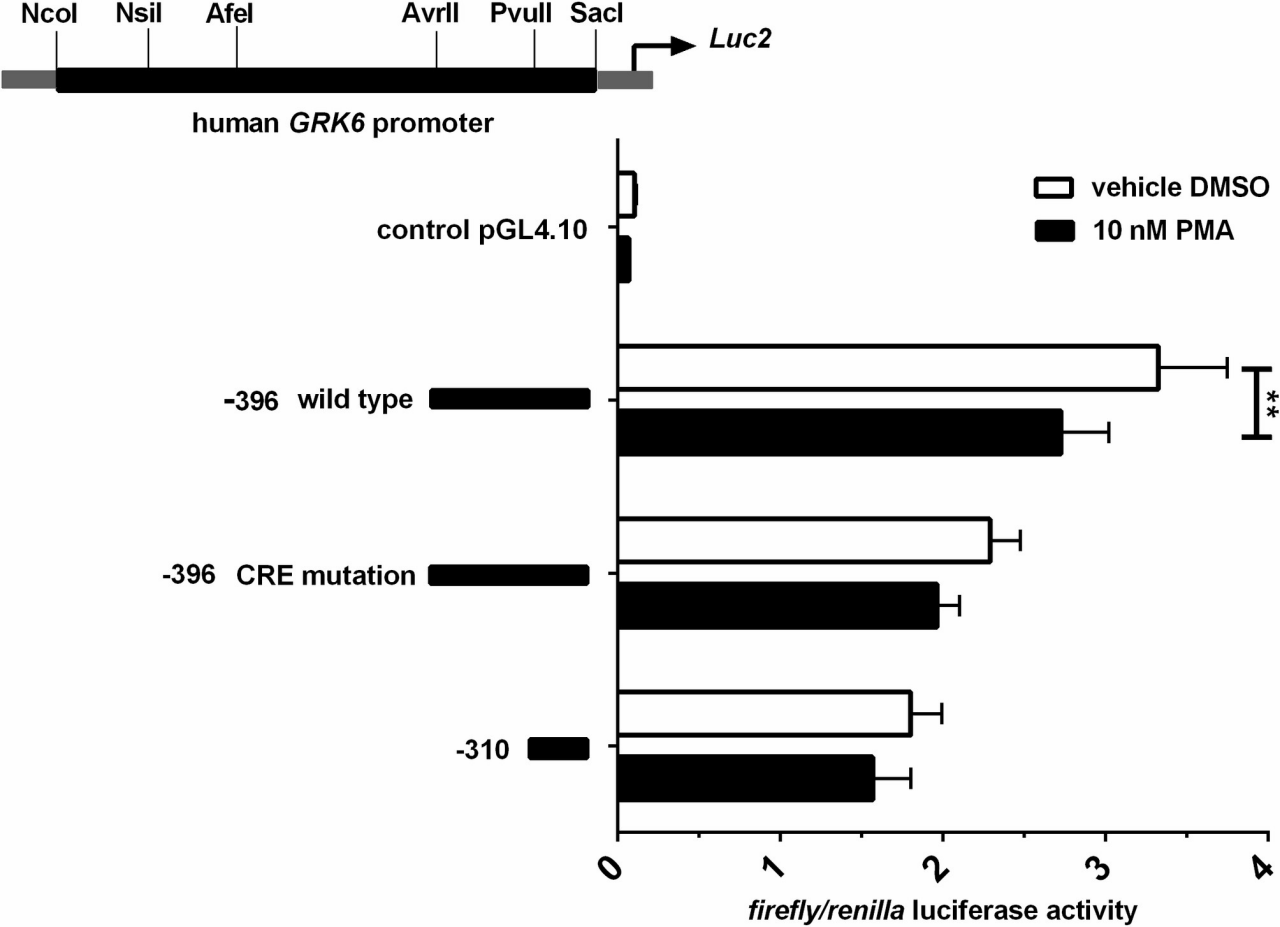
Stimulation with PMA in Jurkat cells led to a decrease in promoter activity. Stimulation was carried out in serum starved cells using 10 nM PMA. Results shown in comparison to vehicle control with DMSO. N = 3, measured in duplicate. Repeated measures two-way ANOVA with Sidak’s multiple comparison test. **p = 0.0027.

To investigate CREB binding to its putative binding site within *GRK6* promoter, electrophoretic mobility shift assays were carried out (Fig 5). Nuclear protein was extracted from Jurkat cells two hours after application of PMA to allow for an increase in the amount of potential transcription factors in the nuclear protein fraction prior to extraction. PMA stimulation was chosen in accordance with our finding that PKC activation leads to an alteration in *GRK6* promoter activity (see Fig 6). It was also previously demonstrated that PKC activation via PMA may induce CREB phosphorylation in blood immune cells [24]. Application of fluorescence labelled GRK6 oligonucleotides (marked by an asterisk) revealed several bands (Fig 5, lane 1). Following the addition of competitors in excess of similar unlabelled GRK6 oligonucleotides, containing the original CREB binding site within the *GRK6* promoter, a putative band of CREB DNA complexes (marked by lower arrow) was eliminated (Fig 5, lane 2). This band also vanished after addition of CREB positive control oligonucleotides given in excess (Fig 5, lane 4), but the overall result produced by this competition differs from the band range in lane 2. Conversely, application of CREB negative control oligonucleotides as competitors did not alter the putative core band (Fig 5, lane 3). Despite the difference in band ranges, this finding makes unspecific binding appear rather unlikely. Furthermore, a super shift was ascertainable after addition of an anti CREB-1 antibody (Fig 5, lane 5, super shift marked by upper arrow). This specific band was completely eliminated by competition with unlabelled GRK6 oligonucleotides (Fig 5, lane 6) and unlabelled CREB positive control oligonucleotides (Fig 5, lane 9). Anti CREB-1 antibody also produced a super shift of virtually identical height when using fluorescence labelled CREB positive control oligonucleotides instead of fluorescence labelled GRK6 oligonucleotides as target sequences (Fig 5, lane 7). This super shift was equally abolished by competition with similar unlabelled CREB positive control oligonucleotides given in excess (Fig 5, lane 8). Taken together, CREB positive control oligonucleotides compete, albeit less effectively than GRK6 positive control oligonucleotides, to the fluorescence labelled GRK6 oligonucleotide target sequence.

### Influence of protein kinase C activity on GRK6 expression

To investigate the influence of protein kinase C (PKC) activity on GRK6 expression, promotor activity was investigated by luciferase assays after stimulation with 10 nM PMA as a direct PKC activator in comparison to DMSO as vehicle control (Fig 6). PMA stimulation evoked a decrease by approximately 20% (p = 0.003) in promoter activity of the -396 construct harboring essential regulatory elements, while only a slight but non-significant reduction was detected in the mutated as well as the shorter construct (Fig 6). This result suggests a downward trend in GRK6 expression after activation of PKC.

**Fig 7:**
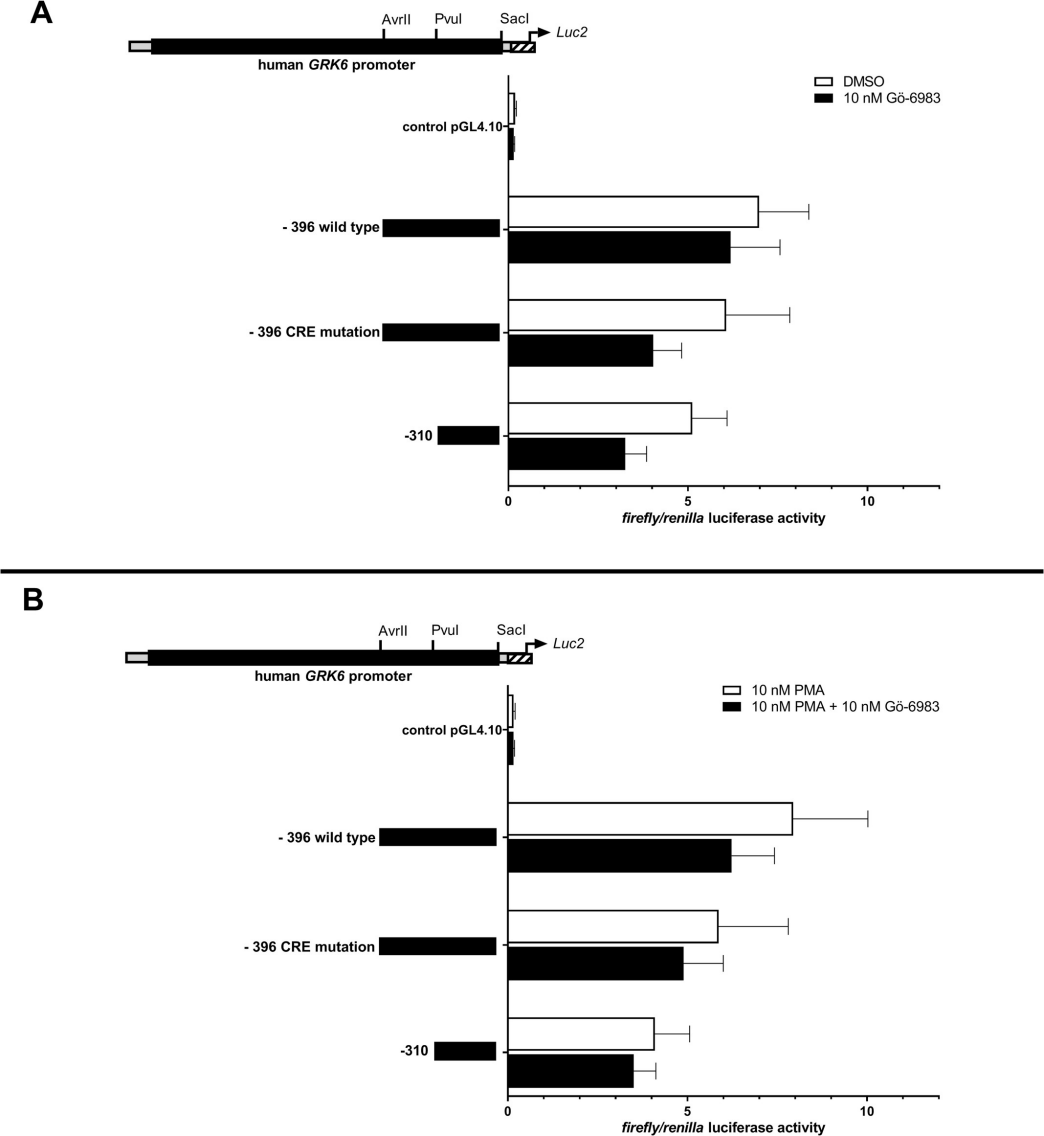
Treatment with pan-PKC inhibitor Gö-6983 resulted in a non-significant decrease in *GRK6* promoter activity. (A) *GRK6* promoter activity after stimulation with 10 nM Gö-6983 in comparison to vehicle control DMSO. Promoter activity was decreased after inhibition of PKC. (B) Combined treatment with 10 nM Gö-6983 + 10 nM PMA in comparison to a control with 10 nM PMA also led to a reduction in *GRK6* promoter activity. Treatment in serum starved Jurkat cells, n = 4, measured in duplicates/triplicates respectively. Depicted are means and standard errors of the mean. Statistics: Ordinary two-way ANOVA and Sidak’s multiple comparisons test.

Interestingly, the treatment of Jurkat cells with 10 nM of a pan-PKC inhibitor (Gö-6983) under similar conditions led to a remarkable but non-significant down regulation of GRK6 promoter activity (Fig 7A), which also persisted in the presence of PMA (Fig 7B).

**Fig 8:**
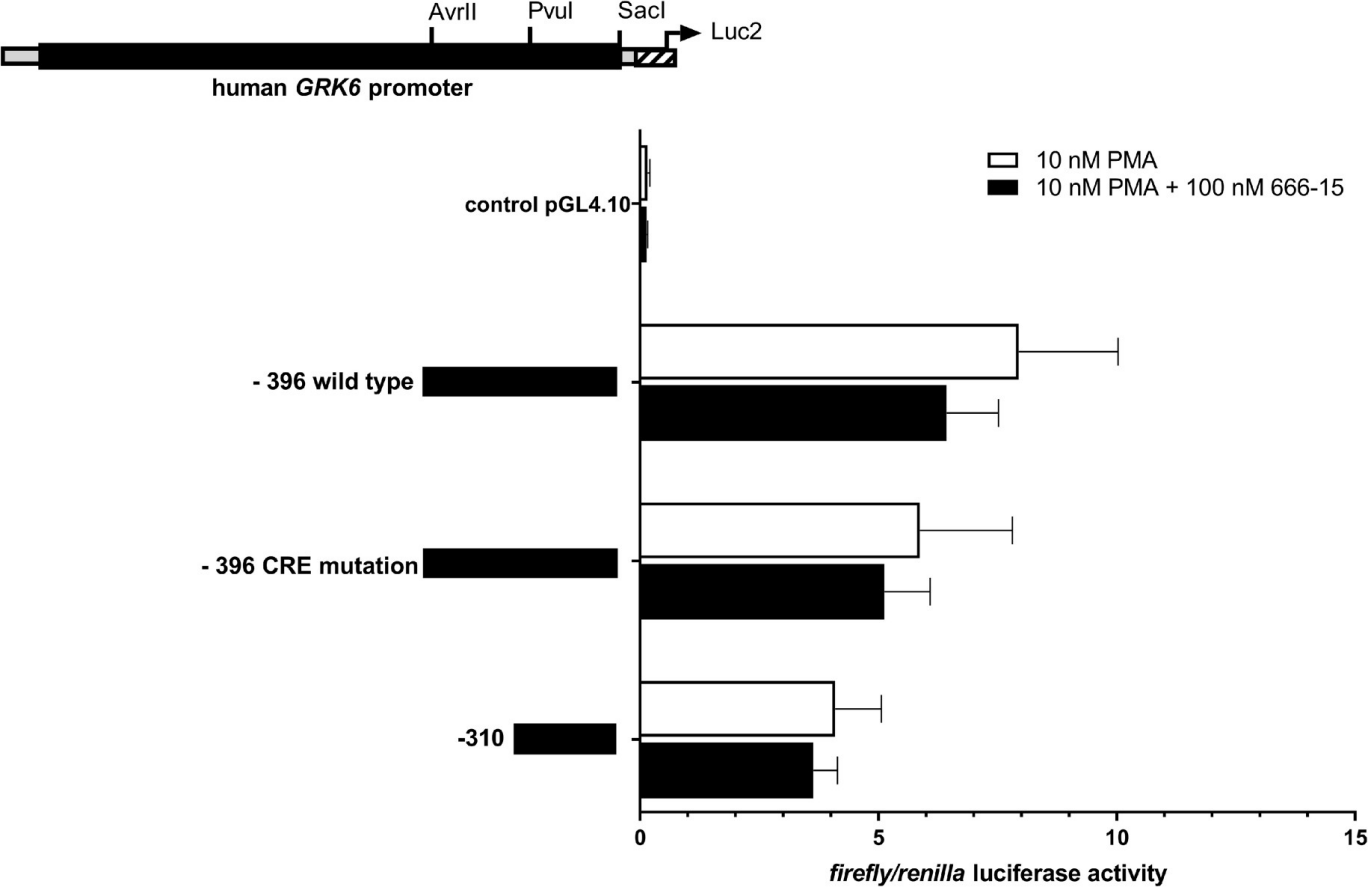
Inhibition of CREB via 666–15 in the presence of PMA resulted in a further decrease in *GRK6* promoter activity. Treatment with 100 nM CREB inhibitor 666–15 + 10 nM PMA in serum starved Jurkat cells, n = 4, measured in duplicates/triplicates respectively. Depicted are means and standard errors of the mean. Statistics: Ordinary two-way ANOVA and Sidak’s multiple comparisons test.

To enquire a potential correlation of the putative CREB binding site in construct– 396 and the effect of PMA stimulation on *GRK6* promoter regulation, we treated the promoter constructs of interest with a combination of PMA und CREB inhibitor 666–15 in Jurkat cells (Fig 8). This constellation produced a minor decline in *GRK6* promoter activity in all constructs investigated in comparison to PMA treatment.

**Fig 9:**
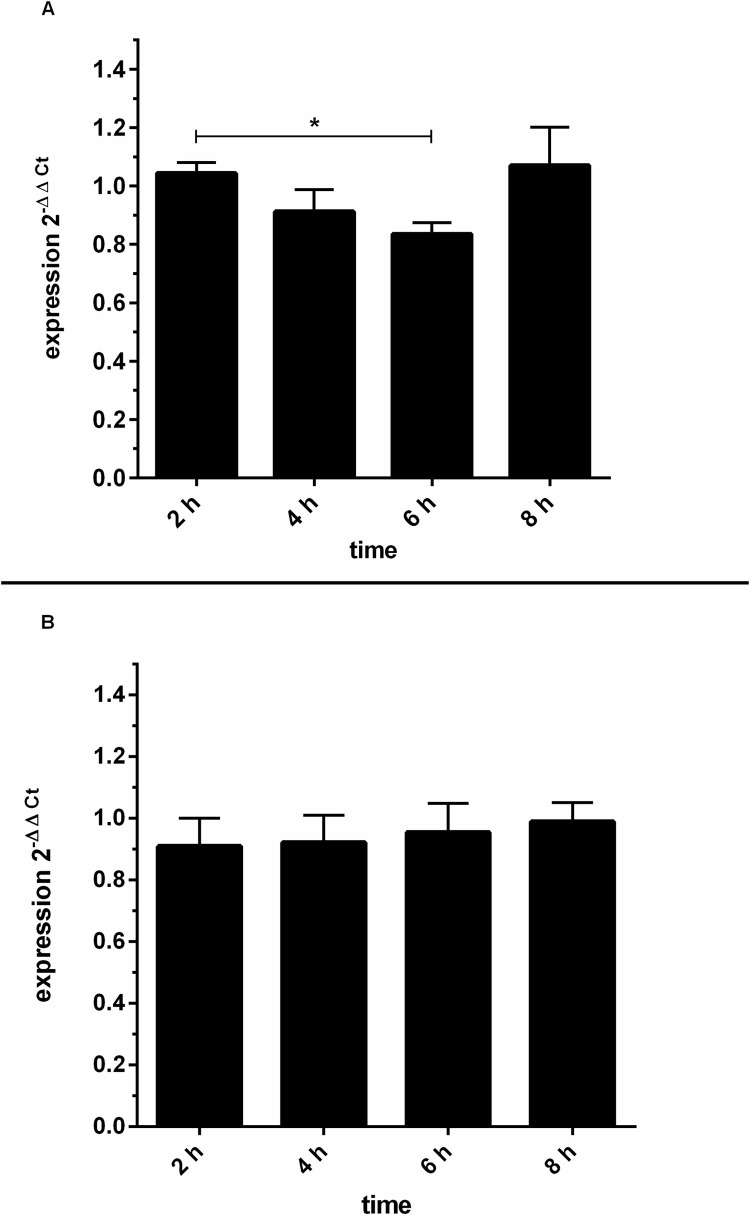
(A) PMA stimulation evoked a slight decrease in endogenous GRK6 mRNA expression after 6 hours. After stimulation with 100 nM PMA, endogenous GRK6 mRNA expression was detected using the comparative 2^-ΔΔCt^ method and vehicle control with DMSO. Over time, mRNA expression considerably decreased after 6h of stimulation. Reference genes used were HPRT and B2M. N = 7, duplicate measurements, one-way repeated measures ANOVA with Bonferroni’s multiple comparisons test. *p = 0.04. (B) Endogenous GRK6 mRNA expression remained unchanged after DMSO treatment. Time course of GRK6 mRNA expression, evaluated by qPCR using the 2^-ΔΔCt^ method, comparing DMSO vehicle control to untreated negative controls. Measurements were carried out in Jurkat cells after serum withdrawal for 2, 4, 6, and 8 hours. N = 7, duplicate measurements.

Furthermore, to investigate GRK6 mRNA expression after stimulation with 100 nM PMA, quantitative real-time PCR was carried out and mRNA expression was observed over a time course of 8 hours (Fig 9). The appropriate concentration of PMA for cell culture stimulations especially for the purpose of investigation of endogenous mRNA expression upon PMA stimulation was tested in various concentrations in Jurkat cells and used in different cell lines in previous works of our group [25]. The concentration of 100 nM PMA led to satisfiable results and PMA concentration was accordingly transferred to the investigation of Jurkat cell cultures.

In accordance with decreased promoter activity in Jurkat cells, endogenous GRK6 mRNA expression was also decreased, in particular after 4 and 6 hours of stimulation, but returned completely to baseline after 8 hours following stimulation (Fig 9A, p = 0.04). mRNA expression decreased by approximately 20% after 6 hours of stimulation. Notably, DMSO as a solvent is also known to increase GRK6 expression [14]. Over a time course of 8 hours, mRNA expression after treatment with DMSO as a vehicle control remained relatively stable in comparison to untreated control cells (Fig 9B). Thus, data distortion through variation caused by stimulation with DMSO can be excluded.

**Fig 10:**
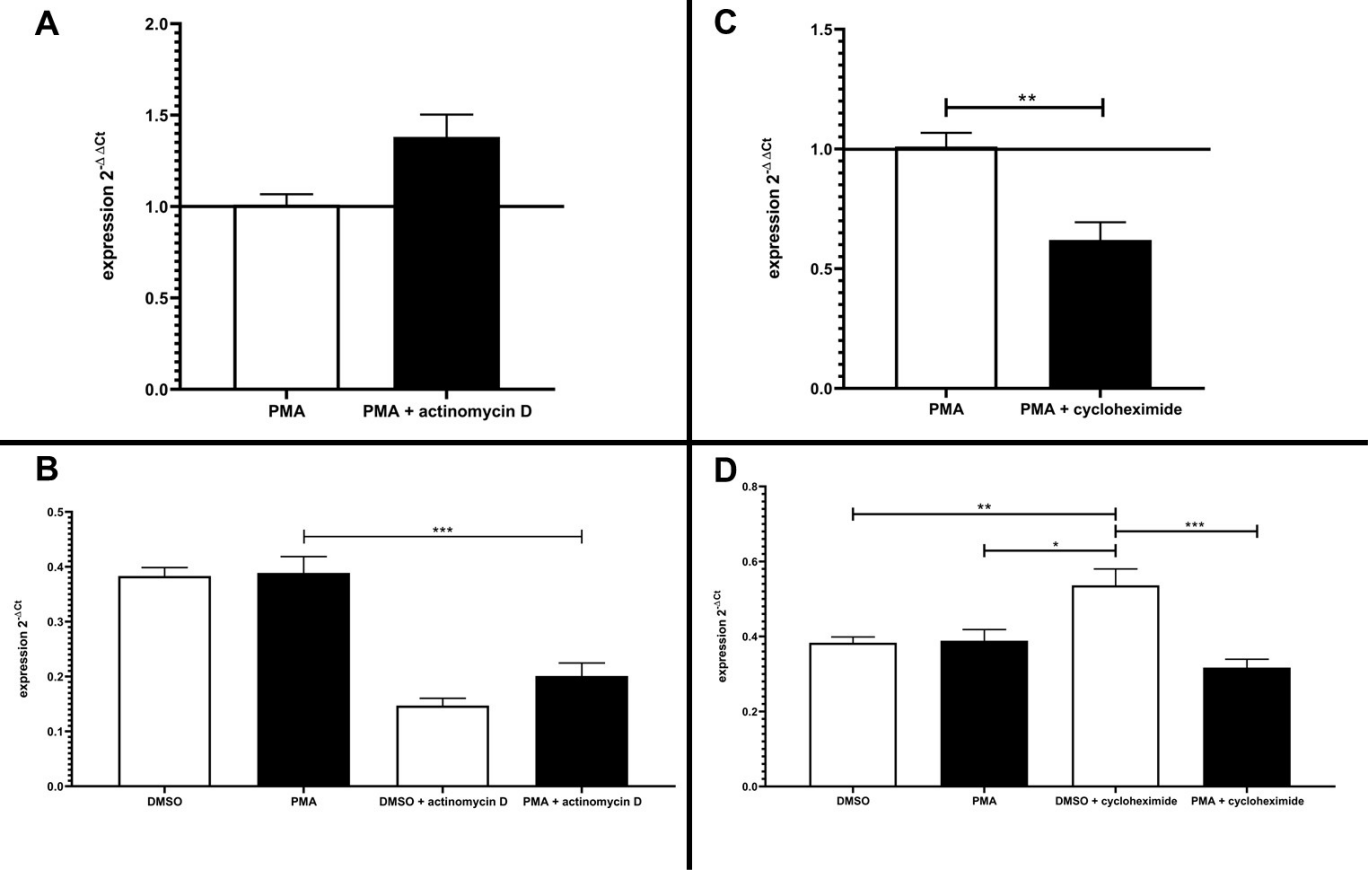
Effects of transcriptional or translational inhibition on GRK6 mRNA expression. (A) Treatment with actinomycin D causing an inhibition of transcriptional activity in serum starved Jurkat cells over 6 hours led to an increase in mRNA expression in comparison to treatment with 100 nM PMA only. Endogenous GRK6 mRNA expression was detected using the comparative 2^-ΔΔCt^ method and vehicle control with DMSO. N = 3, measured in duplicates, mean + standard error of the mean. (B) Depiction of the relative mRNA expression, shown as 2^-Δ Ct^ values in comparison to the reference genes HPRT and B2M. Treatment with actinomycin D resulted in a general decrease in GRK6 mRNA expression. Statistics: Ordinary one-way ANOVA + Sidak’s multiple comparisons test, ***p<0,001. Depicted: mean + standard error of the mean. (C) Inhibition of translational activity by means of cycloheximide over 6h in serum starved Jurkat cells caused a decrease in mRNA expression. GRK6 mRNA expression was calculated using the comparative 2^-ΔΔCt^ method and vehicle control with DMSO. Mann-Whitey-U-test, **p = 0,0043. N = 3, measured in duplicates. Depicted: mean + standard error of the mean. (D) Depiction of the relative mRNA expression, shown as 2^-Δ Ct^ values in comparison to the reference genes HPRT and B2M. Statistics: Ordinary one-way ANOVA + Sidak’s multiple comparisons test. *p = 0,01, **p = 0,008, ***p = 0,0002. Depicted: mean + standard error of the mean.

To exclude distortion of the results due to mRNA instability, Jurkat cells were treated with actinomycin D, which is known to inhibit transcriptional activity (Fig 10A and 10B) [26]. Therefore, a persisting level of mRNA expression under treatment with actinomycin D would indicate a minor contribution of mRNA decay to the reduction of GRK6 mRNA expression observed after stimulation with PMA [27]. Strinkingly, our results showed an increase in GRK6 mRNA expression after treatment with actinomycin D in comparison to treatment solely with PMA (Fig 10A), which is mainly produced by a general decay in mRNA expression after 6h of incubation with actinomycin D in DMSO and PMA treated cells (Fig 10B). Therefore, an unambigious outcome of mRNA stability assays and realtime PCR results regarding PMA stimulation under baseline conditions was not achieved. Nevertheless, treatment with PMA and actinomycin D caused a slight increase in GRK6 mRNA expression in comparison to treatment with vehicle control and actinomycin D only, which might indicate a positive effect on mRNA stabiliziation through PMA treatment (Fig 10A).

Furthermore, endogenous GRK6 mRNA expression was investigated under application of cycloheximide, which is an antibiotic and inhibits protein translation via blocking of the elongation process at the ribosome [28]. Analyses using the comparative 2^-ΔΔCt^ method resulted in a decrease in GRK6 mRNA expression after treatment with cycloheximide and 100 nM PMA in comparison to PMA usage only (Fig 10C) [19]. The depiction of 2^-ΔCt^ values showing the results solely with regard to the reference genes HPRT and B2M points out an increase in GRK6 mRNA expression after stimulation with cycloheximide (Fig 10D). Cycloheximide is known to be a potential transcriptional activator [29]. Under combined administration of cycloheximide and PMA, results remained in comparable ranges to the pre-treatment with DMSO and PMA (Fig 10D), not indicating an increased mRNA decay. Actinomycin D and cycloheximide application also led to a decrease in protein expression, verified by Western blots, according to their characteristics as transcriptional and translational inhibitors (S2 Fig). All in all, mRNA stability cannot be assessed clearly by means of actinomycin D and cycloheximide treatment but is suggestive of balanced changes in mRNA levels under PMA treatment.

**Fig 11:**
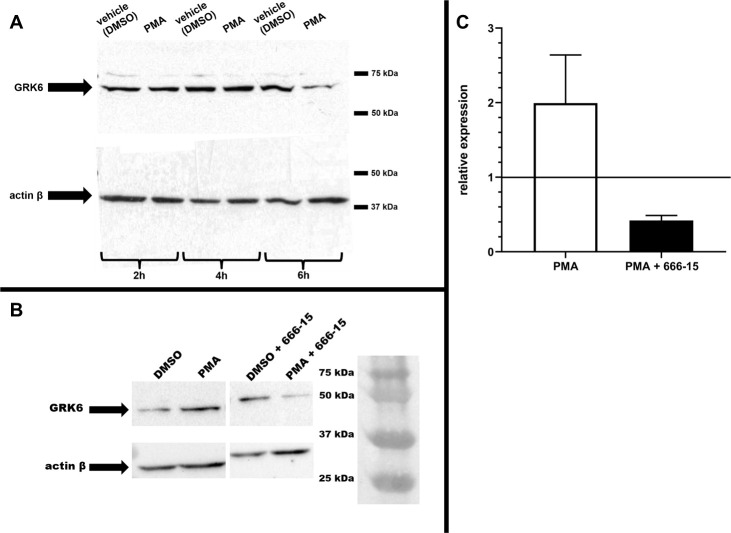
(A) Western blot showed decreased GRK6 protein expression after 6h of PMA stimulation. The main GRK6 band was detected at approx. 70 kDa (molecular weight 66 kDa [21]), a second band at less than 80 kDa. Actin β at approx. 40 kDa (molecular weight 43 kDa) was used as a reference on a stripped membrane. Stimulation with 100 nM PMA led to a distinct decrease in GRK6 protein expression. This figure is cropped. Full-length blots are represented in S3A and S3B Fig. (B) CREB inhibition using 666–15 resulted in decreased GRK6 protein expression under PMA stimulation. Western blot results after 6h of stimulation with 100 nM PMA, and 100 nM 666–15 respectively, in serum starved Jurkat cells. One representative blot of n = 2. This figure is cropped. Full-length blots are represented in S3C and S3D Fig. (C) Quantification of GRK6 protein expression after stimulation with 100 nM PMA and 100 nM 666–15. Band intensity was detected using the Image Lab 6.1 Software. Data was normalized to the band intensity of actin β as reference protein and depicted in relation to the vehicle control with DMSO (= 1). N = 2. Mean + standard error of the mean. Statistics: Mann-Whitney-U-test.

Western blot showed a decrease in GRK6 protein expression after 6 hours of PMA stimulation, whereas actin β (Entrez gene ID: 60) expression used as a reference remained largely unaltered (Fig 11A). A second band seen in the blot has been described previously and might originate from a different molecular mass of GRK6 due to its modifications, such as posttranslational palmitoylation [3, 9, 14] (Fig 11A).

Thus, a decrease in protein expression was observed after 6 hours of PMA stimulation in comparison to loading control (Fig 11A). The inhibition of CREB using 666–15 resulted in a decrease in GRK6 protein expression in combination with PMA stimulation in comparison to PMA stimulaton only (Fig 11B). Visualisation of the data using band intensity measurements and quantification in relation to the reference protein actin β and the vehicle control DMSO produced a fold change of 0,42 after six hours of stimulation with 666–15 (Fig 11C). Remarkably, treatment with 100 nM PMA for six hours in this investigation led to doubling of GRK6 protein expression in comparison to DMSO control (Fig 11C). The reduction in GRK6 protein expression after treatment with 666–15, however, might be an indication that CREB mediated pathways contribute to PMA-induced GRK6 regulation.

## Discussion

Here we characterize the human *GRK6* promotor and identify a CREB binding site within the promoter, impacting on *GRK6* transcriptional regulation. This binding site is located at position -356 to -348 in relation to the translation starting point.

cAMP-responsive element binding protein 1 is a transcription factor binding to a promoter region containing the sequence 5’-TGACGTCA–3’, also designated as cAMP responsive element (CRE). CRE sequences are found in promoters of a broad range of genes. Chiefly, CREB is activated via phosphorylation of serine-133, and various conditions and pathways are known to result in serine-133 phosphorylation, e. g. via the protein kinases A and C. Phosphorylated CREB interacts with its cofactors CREB-binding protein (CBP) or p300 forming a complex supporting the transcription of cAMP-responsive genes as an enhancer. Apart from these processes, several alternative CREB phosphorylation sites and other post-translational modifications were investigated, partly influencing gene transcription in an inhibitory manner (reviewed in [30]).

CREB seems to be involved in the regulation of inflammatory processes by its influence on the expression of immune-related mediators and cytokines (reviewed in [31]). An increase in phosphorylated CREB concentration, for instance, was observed in rat spinal dorsal horns after chronic nerve constriction injury and accompanied by thermal hyperalgesia [32].

Given that the CREB binding site found within the *GRK6* promoter is of functional significance, GRK6 could be one of multiple inflammation related proteins subject to CRE-sequence based transcription processes.

In the context of classical homologous receptor desensitization, GRK proteins take part in negative feedback loops regulating membrane receptor signalling (reviewed in [7]). GRK6 is a regulator of GPCR signals, such as β_2_-adrenergic or D1 receptor signalling [33, 34]. In case of an extensive G_s_-coupled receptor activation, intracellular cumulation of cAMP might lead to a CRE-sequence controlled upregulation of GRK6 expression on a transcriptional level. Since intracellular GRK6 expression levels were shown to be decreased under inflammatory conditions and GRK6 deficiency seems to contribute to hyperalgesia evoked by inflammatory mediators, pharmacologically induced GRK6 upregulation might be a future approach for the treatment of chronic inflammatory diseases [9, 35]. However, it has to be taken into consideration that the complex and not entirely understood interactome of GRKs permits a broad range of potential interactions apart from a linear negative feedback model, which were not further determined in our study [36].

Furthermore, we could show a tendency for GRK6 downregulation on a transcriptional, mRNA, and protein level in response to PMA stimulation, which is known to activate protein kinase C. Although it is questionable whether these changes are solely due to PKC activation, our findings are consistent with previous studies showing the effects of PKC activation on GRK6 expression in a human acute myeloid leukaemia HL-60 cell line [14]. In contrast, in this precedent study, PMA stimulation in primary peripheral blood lymphocyte cell cultures did not result in decreased, but increased GRK6 mRNA and protein levels [14]. One possible reason for this ambiguous behaviour of GRK6 expression levels upon PKC activation might be a differential regulation of protein expression during myelomonocytic cell development [14]. Equivocal behaviour of GRK6 protein and mRNA expression upon PMA stimulation was also observed in our findings (Figs 10, 11B and 11C). Since Jurkat and HL-60 cell lines both arise from leukaemic patients, and thus, results leading to decreased GRK6 expression upon PMA stimulation were achieved in immortalized and pathologically altered cell lines, the assignability of these findings with regard to cell behaviour under physiological conditions *in vivo* is debatable and should be validated in further investigations based on primary cells.

Basically, PKC is a superordinate serine-threonine kinase, which in turn can be allocated to conventional (PKC-α, -β, and -γ), novel (PKC-ɛ, -ɳ, -ɗ, and -θ) and atypical kinases (PKC-ζ and -ί) plus further PKC-related proteins. Both groups of novel and conventional protein kinases C require the presence of phosphatidylserine and diacylglycerol for their activation and are susceptible to phorbol esters, such as phorbol 12-myristate 13-acetate (PMA) (reviewed in [15]). Members of the PKC group act as key regulators of many intracellular conditions, including the mediation of inflammatory processes (reviewed in [15]). It, therefore, can be assumed that PKC may be a signalling molecule via which a decrease in GRK6 expression could be mediated in the context of inflammation.

### Limitations

There are limitations of the present study.

First, the basic presence of a CRE sequence in a promoter does not permit the conclusion of its factual regulation by the transcription factor CREB. Epigenetic modifications influencing the transcription of a CRE sequence to a certain extent have to be taken into account [37]. In addition, CREB proteins are subject to a multitude of potential posttranslational modifications that may regulate the subsequent transcription both in a supportive or inhibitory manner [30]. These aspects were beyond the scope of our current study.

By analysing the results of the EMSA blots, one must consider that there was an apparent difference in competition efficiency between *GRK6* oligonucleotides and CREB positive control oligonucleotides. This difference in binding specificity and competition efficiency of CREB and *GRK6* oligonucleotides may arise from the different nucleotide structure of flanking bases to the core sequence of interest. In fact, the putative CREB binding site within the *GRK6* oligonucleotide contains a base exchange from cytosine to guanine, which might be another reason for less effective binding of CREB oligonucleotides to the fluorescence labelled *GRK6* target sequence. Despite this discrepancy in binding efficiency, however, the results clearly indicate a specific binding of CREB to *GRK6* oligonucleotides regarding the putative core band marked by the lower arrow in the blot and, therefore, support the hypothesis of a functional CRE sequence in the *GRK6* promoter.

Albeit, CREB inhibition using 666–15 did not lead to a clear reduction in *GRK6* promoter activity, especially in construct -396, containing the CREB binding site investigated (Fig 4). Upon combined treatment with 666–15 and PMA, *GRK6* promoter activity was slightly reduced in all constructs investigated, even after mutation of a CREB binding site (Fig 8). It is conceivable that multiple CREB sensitive regulatory elements do exist in the *GRK6* promoter. A thorough investigation of our shortest *GRK6* promoter construct -310 might help to shed light onto this question.

Notably, PMA is a general activator of PKC, except for atypical PKC isoforms [38]. Therefore, a multitude of PKC isotypes may cause the results shown in this study. A distinct differentiation by which PKC isoform GRK6 downregulation was initiated, is not yet possible. Besides, phorbol esters such as PMA, do not exclusively activate PKC but also several other mediators containing a phorbol ester sensitive C1 domain. For instance, diacylglycerol kinases, chimaerins and small GTPases represent only a small subset of proteins that are equally inducible by phorbol esters [39]. A potential influence of these mediators on GRK6 expression and on our results cannot be entirely excluded.

Regarding the ambiguous results following stimulation with PMA in monomyeloic cell differentiation [14], which were also detectable in Jurkat cells in our findings, further investigations including differential cell lines, unique, protein kinase C subtype specific stimulants and a close-meshed investigation of various stimulation periods are imperative to assess the circumstances of GRK6 up- or downregulation upon protein kinase C activation. Also, a further decrease in GRK6 promoter activity upon inhibition of protein kinase C using Gö-6983 (Fig 7) does not allow to draw concrete conclusions about GRK6 regulation regarding protein kinase C activity.

Besides, another GRK isoenzyme, GRK2, which is also expressed ubiquitously, was recently described to contribute to sustained CREB activation in neuronal cells [40]. To date, it is still unclear whether GRK2 is capable of directly phosphorylating CREB or whether this process is mediated through interposed regulatory pathways [40]. In this context, a GRK2-regulated CREB activation in the *GRK6* promoter might be conceivable. An inter-GRK controlled regulation of expression could represent a captivating field for further investigations, which may contribute to a more extensive understanding of the GRK interactome.

Results of realtime PCR detection of endogenous GRK6 mRNA expression should be interpreted cautiously since there are several reasons for the decrease seen in endogenous mRNA levels after actinomycin D stimulation. At first, the reduced level of mRNA might be a result of general instability and decay of GRK6 mRNA. Furthermore, it could also be caused by cytotoxic effects of actinomycin D, especially due to long term treatment of Jurkat cells with actinomycin D over 6 hours [28], and thus, reduced cell viability culminating in a decrease in overall mRNA expression. It would be useful to further examine the effects of actinomycin D treatment in a course considering numerous points in time and multiple mRNA targets as well as shorter intervals of actinomycin D treatment in Jurkat cells. To properly distinguish the underlying causes of mRNA decay, investigations including different assays to monitor mRNA stability, as described elsewhere [41], could be helpful.

## Conclusion

In conclusion, we characterized the *GRK6* promoter and found an activity determining region at position -348 to -356 within the *GRK6* promoter containing a functional CREB binding site. Specific interaction of CREB and the putative CRE sequence in the *GRK6* promoter was confirmed by EMSA. Furthermore, a tendency in downregulation of GRK6 expression was revealed on the transcriptional, mRNA, and protein levels, presumably protein kinase C mediated. The results of this study may suggest a potential contribution of PKC related signalling pathways to *GRK6* regulation in inflammatory cells.

## Supporting information

S1 Fig (A-C) Uncropped EMSA blot outlined a specific CREB binding site. (A) Each lane contains 5 or 4 μg of nuclear protein extracted from Jurkat cells as indicated. Fluorescence labelled GRK6 oligonucleotides (GRK6*, lanes 1–7, 10–12 and 15) or CREB1 positive controls (CREB positive*, lanes 8, 9, 13, 14), marked by an asterisk, were used for binding detection. Unlabelled oligonucleotides, either containing the underlying GRK6 sequence (*GRK*, lanes 3, 7 and 12), CREB1 positive control (abbreviated to *C*, lanes 5, 9, 10, 14, 15) or CREB1 negative control (abbreviated to *C neg*, lane 4) without suitable binding sites, were applied in 200 times excess as competitors to unravel potential unspecific binding. Concerning the super shift, 2 μg of anti CREB-1 antibody were added per lane (lanes 6–15) to verify specific binding. Lanes marked with “X” are not shown in the article. (B) Each lane contains 5 μg of nuclear protein extracted from Jurkat cells as indicated. Fluorescence labelled GRK6 oligonucleotides (GRK6*, lanes 6–10), CREB1 positive control (CREB positive*, lanes 1–4), CREB1 negative control (CREB negative*, lane 5), and GRK6 M1 oligonucleotide containing the mutated CREB binding site as outlined in Fig 3A (GRK6 M1*, lanes 11–15) were used for binding detection. Unlabelled oligonucleotides were applied in 200 times excess as competitors: CREB1 positive control (abbreviated to C, lanes 3, 10, 15), CREB1 negative control (abbreviated to C neg, lanes 4 and 9), GRK6 positive control (GRK, lanes 8 and 14), GRK6 M1 (GRK M1, lane 13). (C) Each lane contains 4 μg of nuclear protein extracted from Jurkat cells. Fluorescence labelled GRK6 oligonucleotides (GRK6*, lanes 6–12, 15), CREB1 positive controls (CREB positive*, lanes 1–4, 13, 14), and CREB1 negative controls (CREB negative*, lane 5) were used for binding detection. Unlabelled competitors were applied in 200 times excess as follows: CREB1 positive control (abbreviated to C, lanes 3, 10, 14, 15), CREB1 negative control (abbreviated to C neg, lanes 4, 9), and GRK6 positive control (abbreviated to GRK, lanes 8, 12). Concerning the super shift, 2 μg of anti CREB-1 antibody were added per lane (lanes 11–15) to verify specific binding. Super shift was marked by an arrow as indicated.
(PDF)

S2 Fig (A) Inhibition of translation and transcription by means of actinomycin D and cycloheximide led to a decrease in GRK6 protein expression detected in Western blot assay. The main GRK6 band was detected at approx. 70 kDa (molecular weight 66 kDa [22]). Actin β at approx. 40 kDa (molecular weight 43 kDa) was used as reference protein on a stripped membrane. Stimulation with 100 nM PMA, actinomycin D (5 μg/ml) or cycloheximide (20 μg/ml) was carried out in serum starved Jurkat cells for 6 hours as indicated. DMSO was used as vehicle control. This figure is cropped. Full-length blots are represented in S2B and S2C Fig. (B) Uncropped Western blot of GRK6 protein expression after treatment with actinomycin D and cycloheximide as indicated for six hours in serum starved Jurkat cells. For details see caption S2A Fig. Detection of GRK6 protein at approx. 70 kDa. (C) Uncropped Western blot of actin β protein expression after treatment with actinomycin D and cycloheximide as indicated for six hours in serum starved Jurkat cells. For details see caption S2A Fig. After membrane stripping, actin β was used as reference protein.
(PDF)

S3 Fig (A) Uncropped Western blot of GRK6 protein expression in Jurkat cells. The main GRK6 band was detected at approx. 70 kDa (molecular weight 66 kDa), a second band at less than 80 kDa. Stimulation with 100 nM PMA led to a distinct decrease in GRK6 protein expression after 6 h of stimulation. (B) Uncropped Western blot of actin β protein expression in Jurkat cells. After membrane stripping, actin β at approx. 40 kDa (molecular weight 43 kDa) was used as a reference protein. (C) Uncropped Western blot of GRK6 protein expression in Jurkat cells including stimulation with CREB inhibitor 666–15 (blot 1). Serum starved Jurkat cells were stimulated with 10 nM pan-PKC inhibitor Gö-6983, 60 nM pan-PKC inhibitor Gö-6983 and 100 nM CREB inhibitor 666–15 +/- 100 nM PMA as indicated for 6 hours. GRK6 was detected at approx. 70 kDa. (D) Uncropped Western blot of actin β protein expression in Jurkat cells including stimulation with CREB inhibitor 666–15 (blot 1). Serum starved Jurkat cells were stimulated with 10 nM pan-PKC inhibitor Gö-6983, 60 nM pan-PKC inhibitor Gö-6983 and 100 nM CREB inhibitor 666–15 +/- 100 nM PMA as indicated for 6 hours. Actin β was detected at approx. 40 kDa on a stripped membrane. (E) Uncropped Western blot of GRK6 protein expression in Jurkat cells including stimulation with CREB inhibitor 666–15 (blot 2). Serum starved Jurkat cells were stimulated with 10 nM pan-PKC inhibitor Gö-6983, 60 nM pan-PKC inhibitor Gö-6983 and 100 nM CREB inhibitor 666–15 +/- 100 nM PMA as indicated for 6 hours. GRK6 main band was detected at approx. 70 kDa. (F) Uncropped Western blot of actin β protein expression in Jurkat cells including stimulation with CREB inhibitor 666–15 (blot 2). Serum starved Jurkat cells were stimulated with 10 nM pan-PKC inhibitor Gö-6983, 60 nM pan-PKC inhibitor Gö-6983 and 100 nM CREB inhibitor 666–15 +/- 100 nM PMA as indicated for 6 hours. Actin β was detected at approx. 40 kDa on a stripped membrane.
(PDF)
