# Supplementary material for: Characterization of the G protein-coupled receptor kinase 6 promoter reveals a functional CREB binding site
Source: PLoS One. 2021 Feb 18;16(2):e0247087. doi: 10.1371/journal.pone.0247087 (PMC7891717; doi:10.1371/journal.pone.0247087)

Fig S1 (A)

lane no.	*1*	*2*	*3*	*4*	*5*	*6*	*7*	*8*	*9*	*10*	*11*	*12*	*13*	*14*	*15*
amount of nuclear extract [µg]	---	5	5	5	5	5	5	5	5	5	4	4	4	4	4
GRK6*	+	+	+	+	+	+	+	---	---	+	+	+	---	---	+
CREB positive*	---	---	---	---	---	---	---	+	+	---	---	---	+	+	---
Unlabelled competitor	---	---	GRK	C neg	C	---	GRK	---	C	C	---	GRK	---	C	C
anti CREB-1	---	---	---	---	---	+	+	+	+	+	+	+	+	+	+

super shift

CREB DNA complexes

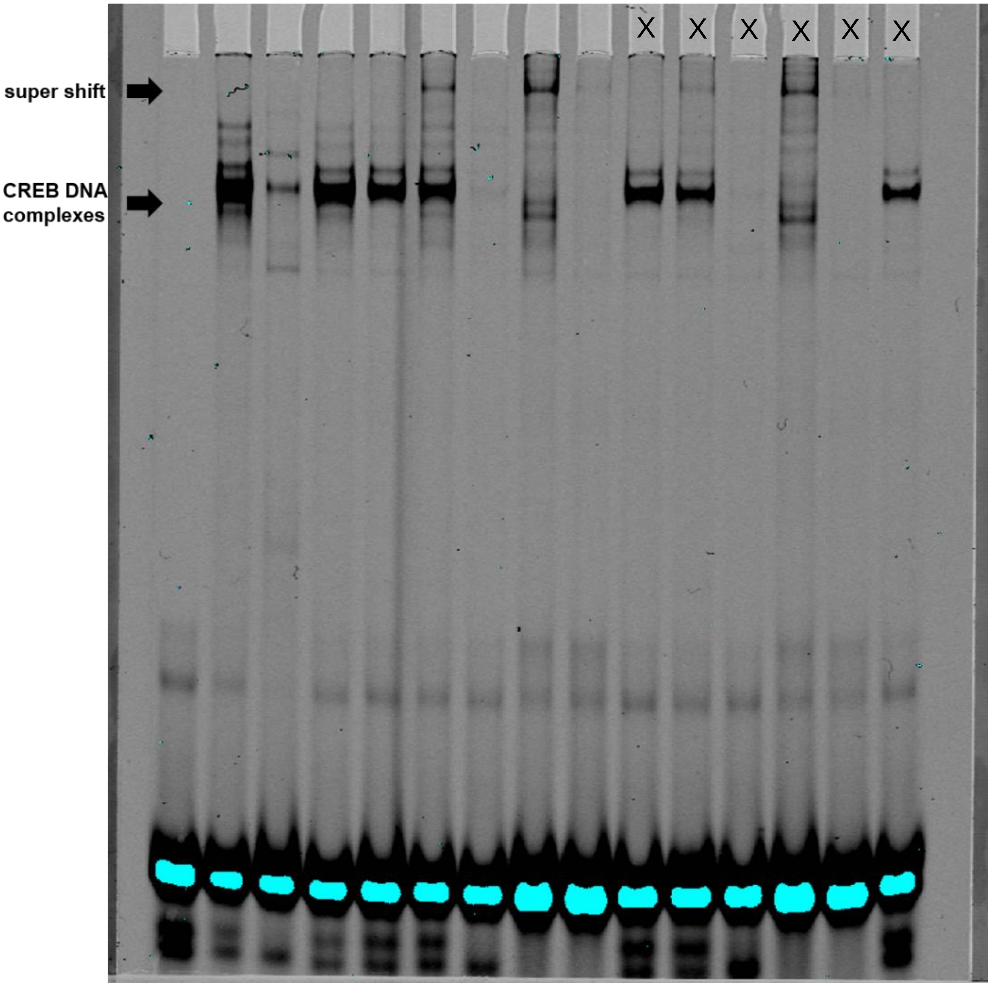

Fig S1 (B)

lane no.	*1*	*2*	*3*	*4*	*5*	*6*	*7*	*8*	*9*	*10*	*11*	*12*	*13*	*14*	*15*
**amount of nuclear extract [µg]**	---	---	5	5	5	---	5	5	5	5	---	5	5	5	5
**GRK6***	---	---	---	---	---	+	+	+	+	+	---	---	---	---	---
**CREB positive***	+	+	+	+	---	---	---	---	---	---	---	---	---	---	---
**CREB negative***	---	---	---	---	+	---	---	---	---	---	---	---	---	---	---
**GRK6 M1***	---	---	---	---	---	---	---	---	---	---	+	+	+	+	+
**unlabelled competitor**	---	---	C	C neg	---	---	---	GRK	C neg	C	---	---	GRK M1	GRK	C

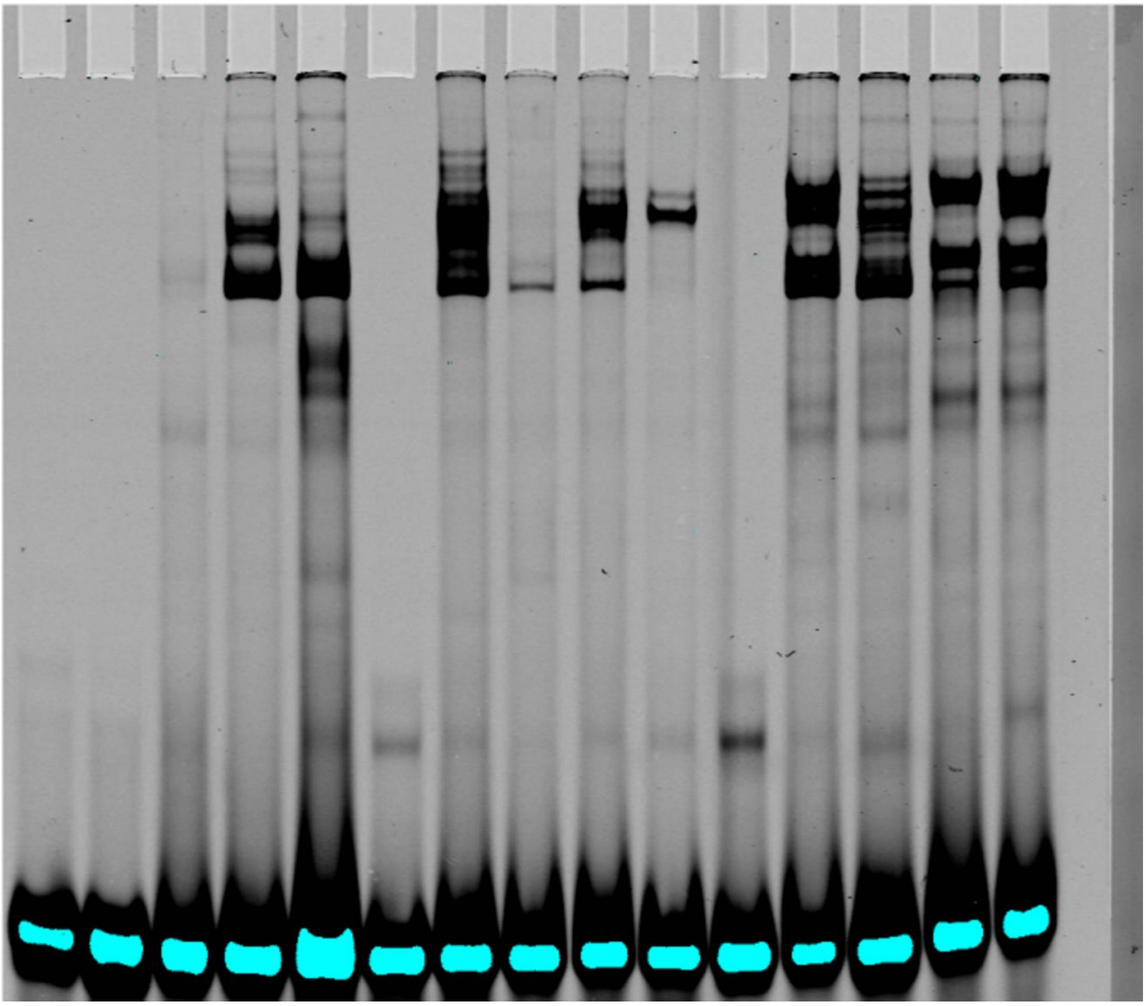

Fig S1 (C)

lane no.	*1*	*2*	*3*	*4*	*5*	*6*	*7*	*8*	*9*	*10*	*11*	*12*	*13*	*14*	*15*
**amount of nuclear extract [µg]**	---	4	4	4	4	---	4	4	4	4	4	4	4	4	4
**GRK6***	---	---	---	---	---	+	+	+	+	+	+	+	---	---	+
**CREB positive***	+	+	+	+	---	---	---	---	---	---	---	---	+	+	---
**CREB negative***	---	---	---	---	+	---	---	---	---	---	---	---	---	---	---
**anti CREB-1**	---	---	---	---	---	---	---	---	---	---	+	+	+	+	+
**unlabelled competitor**	---	---	C	C neg	---	---	---	GRK	C neg	C	---	GRK	---	C	C

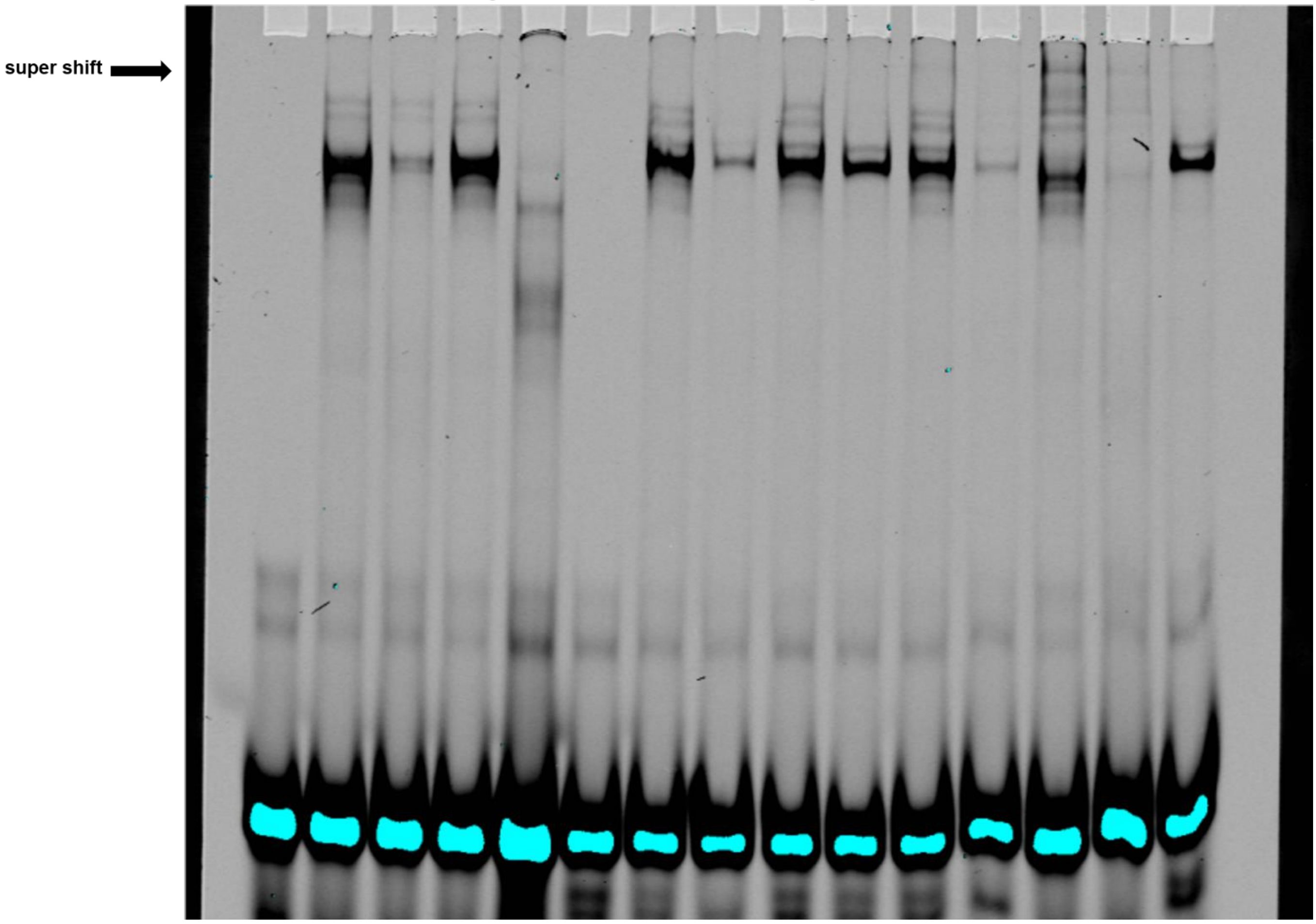

Supplement: S1 Fig — (A-C) Uncropped EMSA blot outlined a specific CREB binding site. (A) Each lane contains 5 or 4 μg of nuclear protein extracted from Jurkat cells as indicated. Fluorescence labelled GRK6 oligonucleotides (GRK6*, lanes 1–7, 10–12 and 15) or CREB1 positive controls (CREB positive*, lanes 8, 9, 13, 14), marked by an asterisk, were used for binding detection. Unlabelled oligonucleotides, either containing the underlying GRK6 sequence (GRK, lanes 3, 7 and 12), CREB1 positive control (abbreviated to C, lanes 5, 9, 10, 14, 15) or CREB1 negative control (abbreviated to C neg, lane 4) without suitable binding sites, were applied in 200 times excess as competitors to unravel potential unspecific binding. Concerning the super shift, 2 μg of anti CREB-1 antibody were added per lane (lanes 6–15) to verify specific binding. Lanes marked with “X” are not shown in the article. (B) Each lane contains 5 μg of nuclear protein extracted from Jurkat cells as indicated. Fluorescence labelled GRK6 oligonucleotides (GRK6*, lanes 6–10), CREB1 positive control (CREB positive*, lanes 1–4), CREB1 negative control (CREB negative*, lane 5), and GRK6 M1 oligonucleotide containing the mutated CREB binding site as outlined in Fig 3A (GRK6 M1*, lanes 11–15) were used for binding detection. Unlabelled oligonucleotides were applied in 200 times excess as competitors: CREB1 positive control (abbreviated to C, lanes 3, 10, 15), CREB1 negative control (abbreviated to C neg, lanes 4 and 9), GRK6 positive control (GRK, lanes 8 and 14), GRK6 M1 (GRK M1, lane 13). (C) Each lane contains 4 μg of nuclear protein extracted from Jurkat cells. Fluorescence labelled GRK6 oligonucleotides (GRK6*, lanes 6–12, 15), CREB1 positive controls (CREB positive*, lanes 1–4, 13, 14), and CREB1 negative controls (CREB negative*, lane 5) were used for binding detection. Unlabelled competitors were applied in 200 times excess as follows: CREB1 positive control (abbreviated to C, lanes 3, 10, 14, 15), CREB1 negative control (abbre [file pone.0247087.s001.pdf]
