# Supplementary figures and images for: Characterization of the G protein-coupled receptor kinase 6 promoter reveals a functional CREB binding site
Source: PLoS One. 2021 Feb 18;16(2):e0247087. doi: 10.1371/journal.pone.0247087 (PMC7891717; doi:10.1371/journal.pone.0247087)

Fig S2 (A)

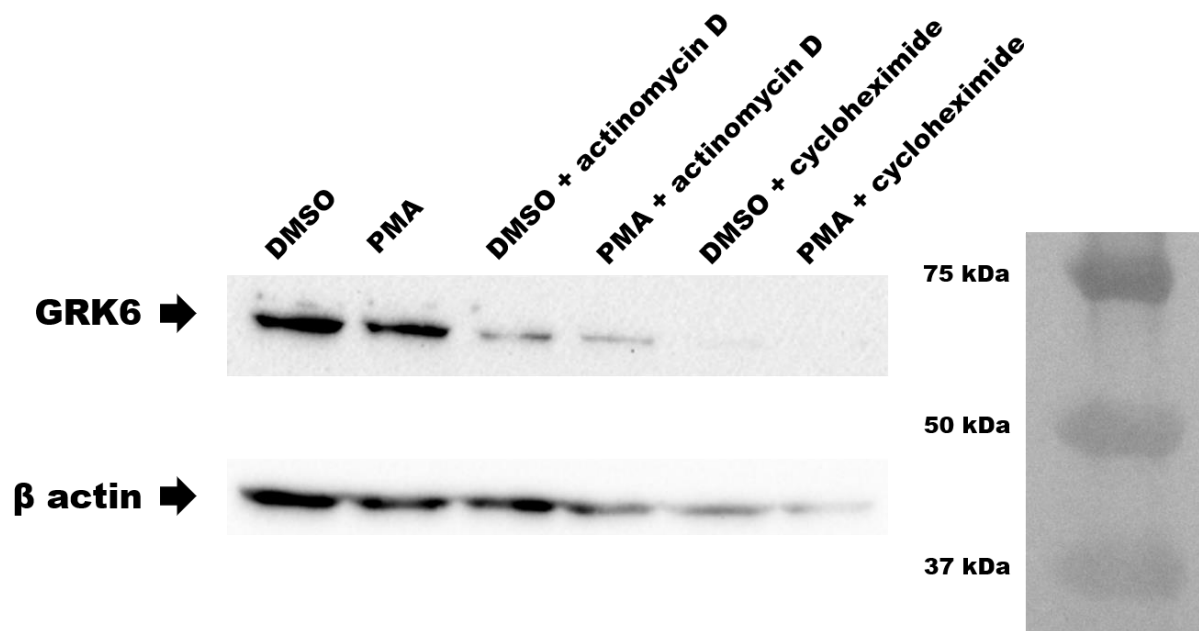

**Fig S2 (B)**

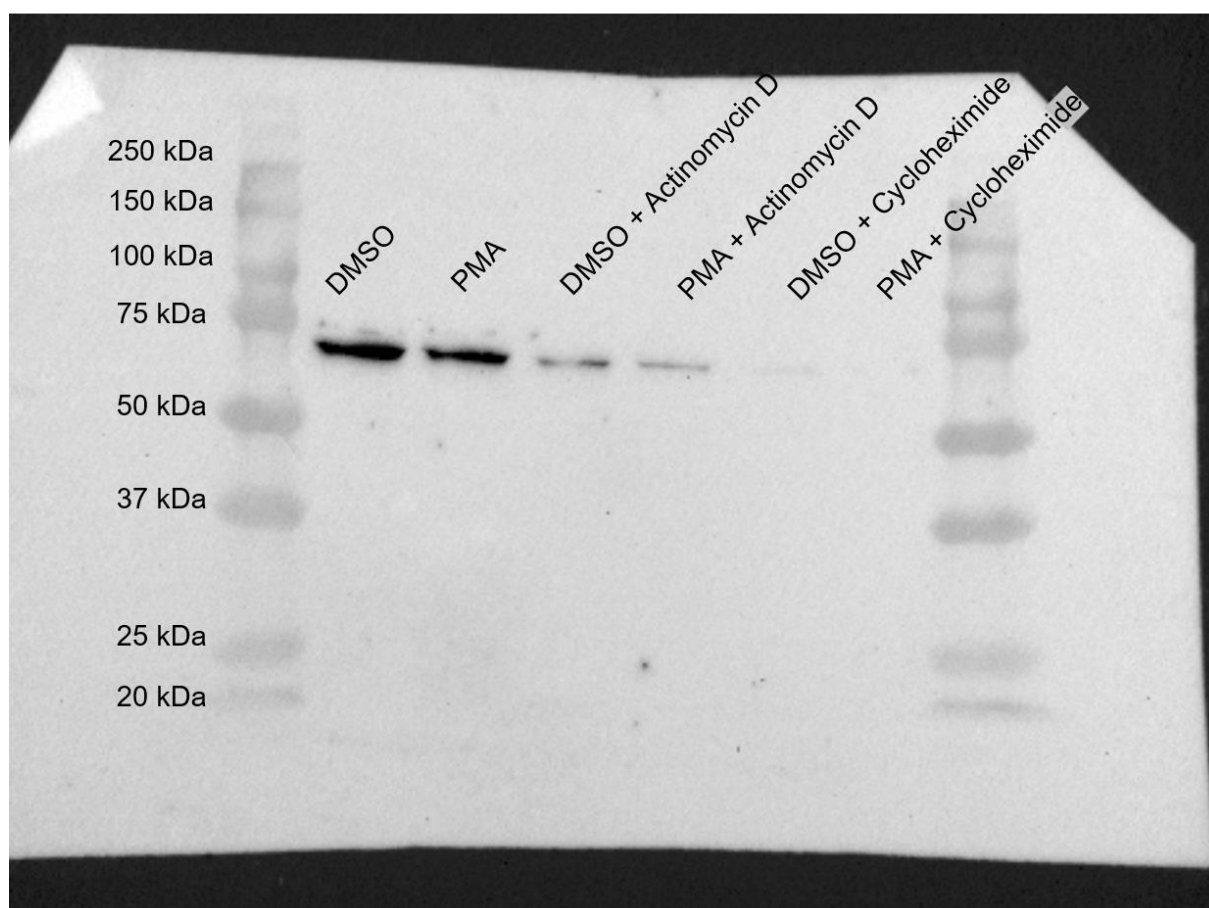

**Fig S2 (C)**

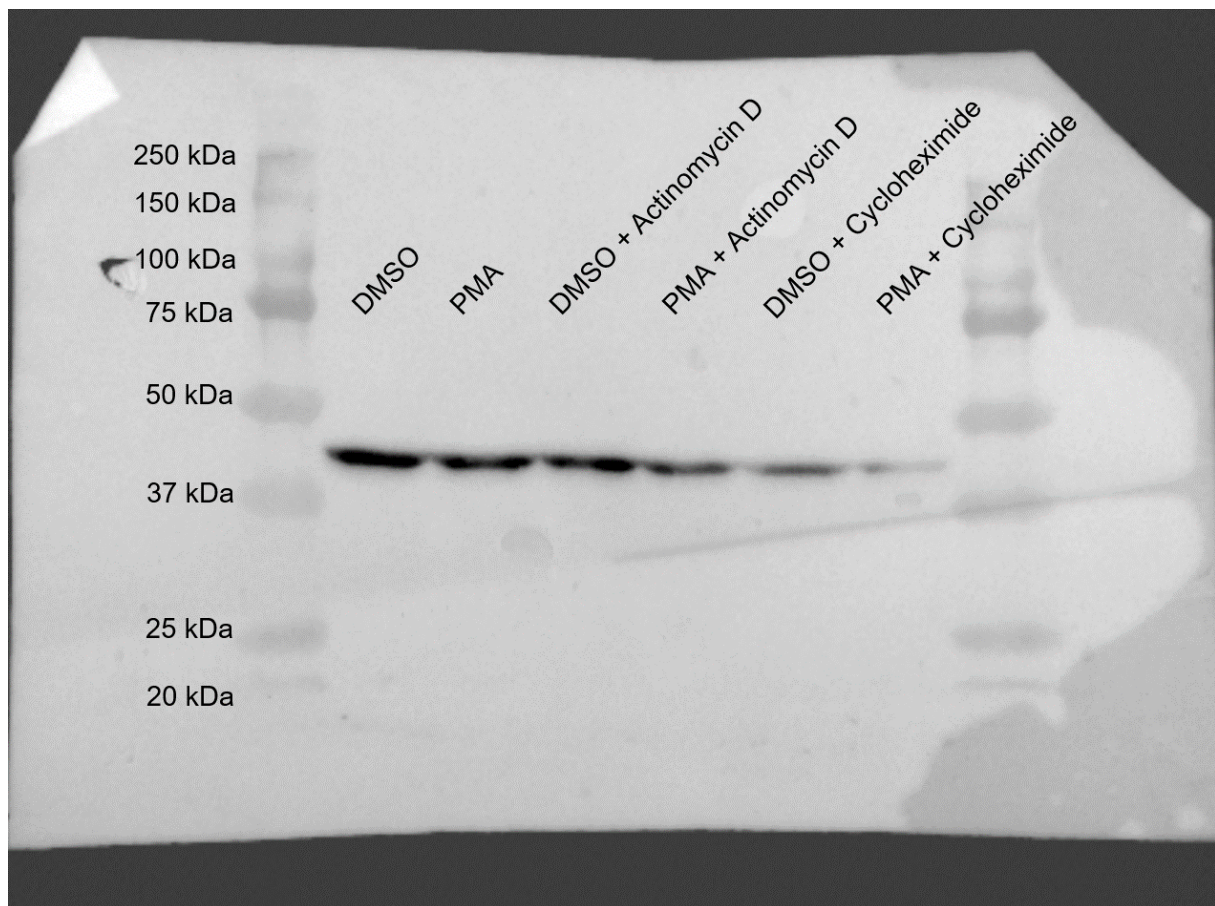

Supplement: S2 Fig — (A) Inhibition of translation and transcription by means of actinomycin D and cycloheximide led to a decrease in GRK6 protein expression detected in Western blot assay. The main GRK6 band was detected at approx. 70 kDa (molecular weight 66 kDa [22]). Actin β at approx. 40 kDa (molecular weight 43 kDa) was used as reference protein on a stripped membrane. Stimulation with 100 nM PMA, actinomycin D (5 μg/ml) or cycloheximide (20 μg/ml) was carried out in serum starved Jurkat cells for 6 hours as indicated. DMSO was used as vehicle control. This figure is cropped. Full-length blots are represented in S2B and S2C Fig. (B) Uncropped Western blot of GRK6 protein expression after treatment with actinomycin D and cycloheximide as indicated for six hours in serum starved Jurkat cells. For details see caption S2A Fig. Detection of GRK6 protein at approx. 70 kDa. (C) Uncropped Western blot of actin β protein expression after treatment with actinomycin D and cycloheximide as indicated for six hours in serum starved Jurkat cells. For details see caption S2A Fig. After membrane stripping, actin β was used as reference protein. (PDF) [file pone.0247087.s002.pdf]

Fig S3 (A) + (B)

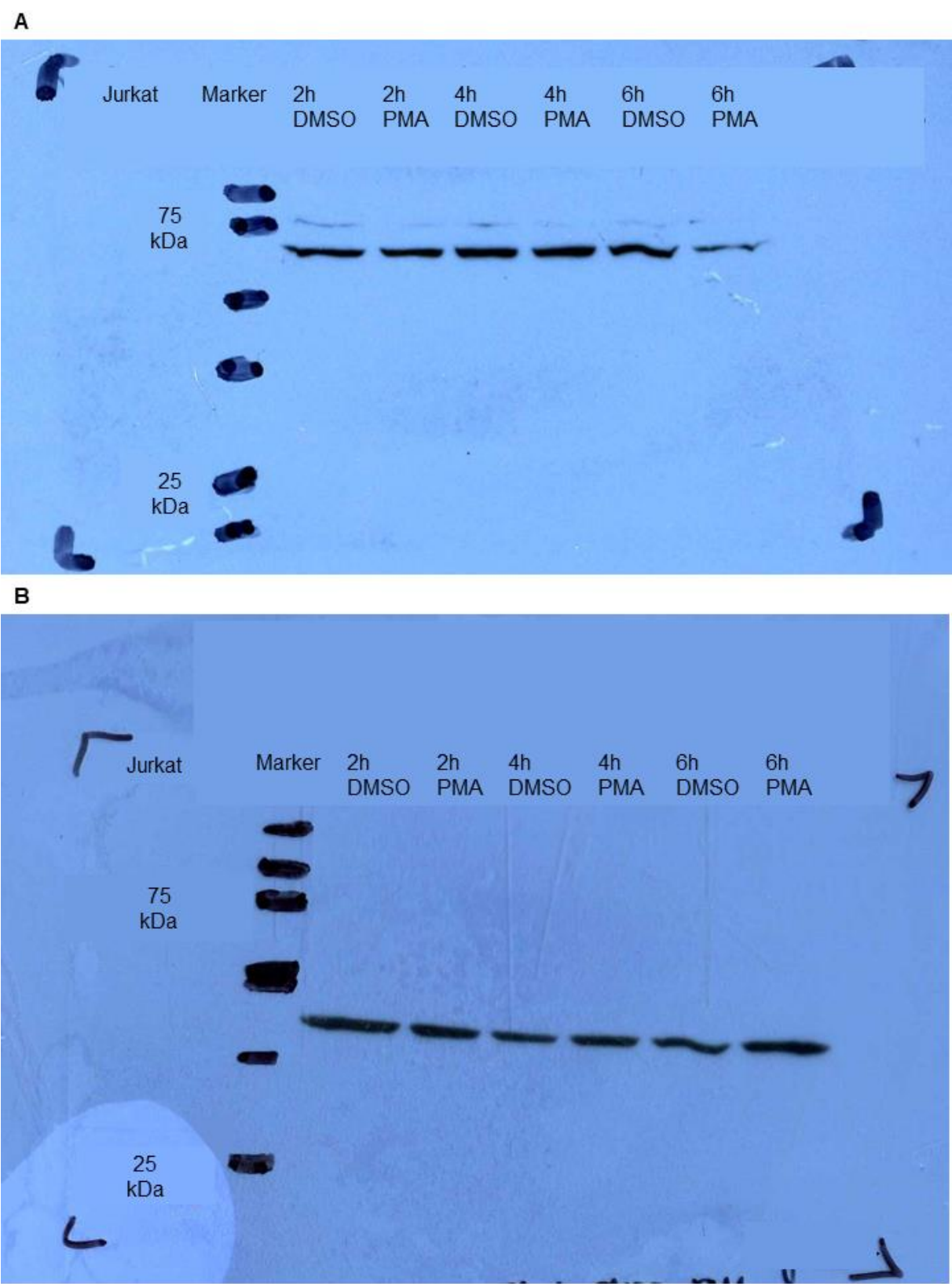

**Fig S3 (C)**

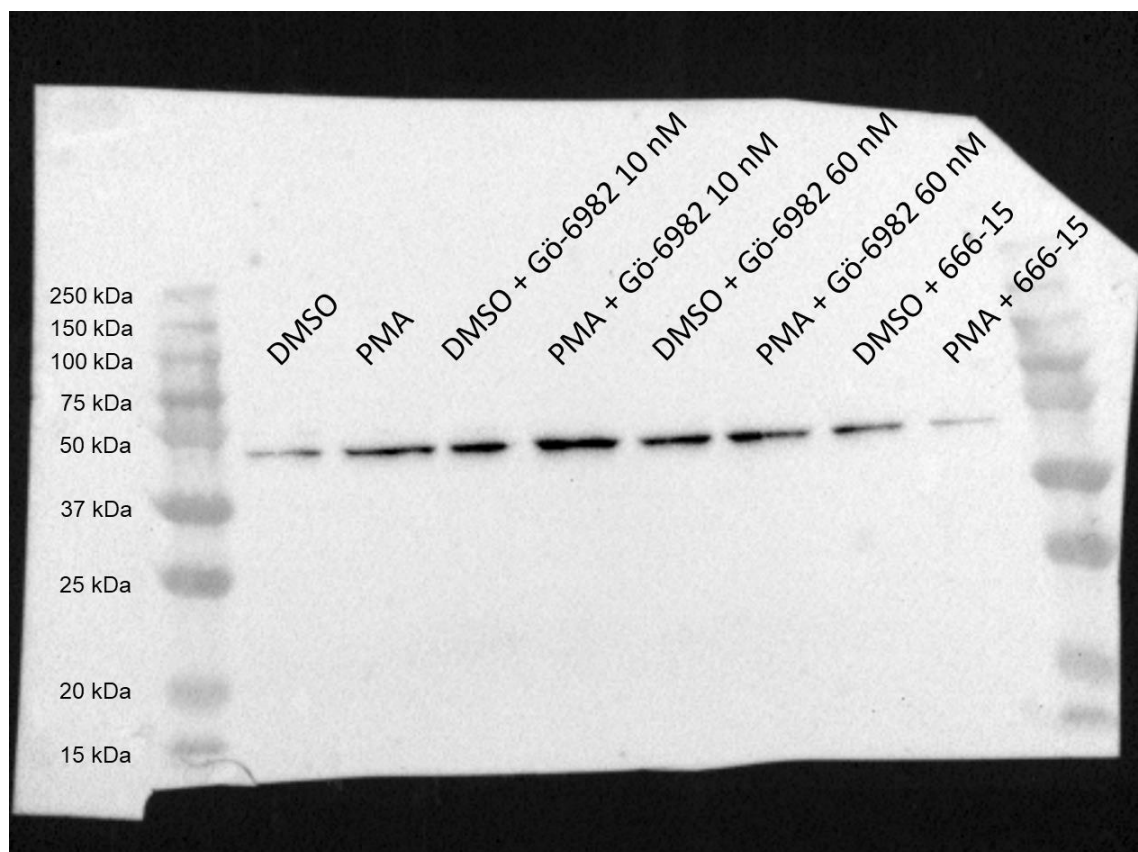

**Fig S3 (D)**

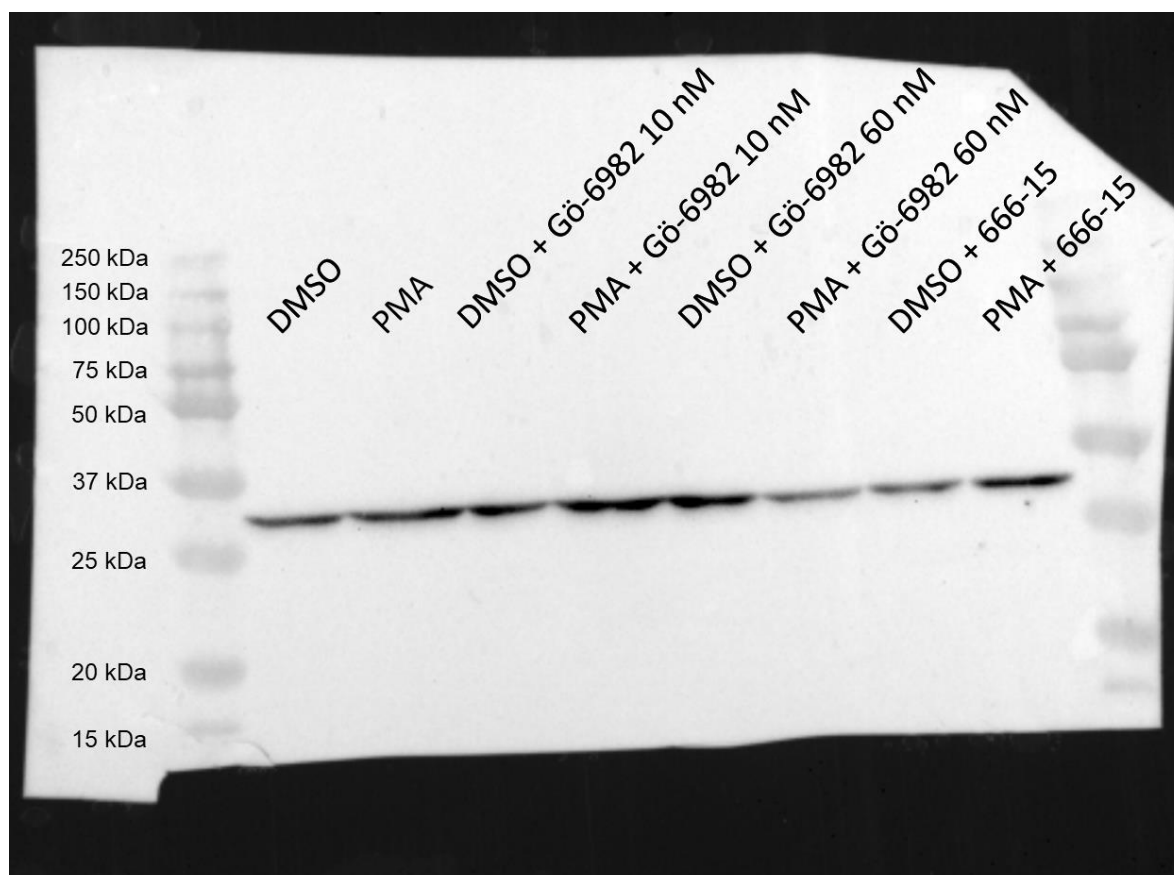

**Fig S3 (E)**

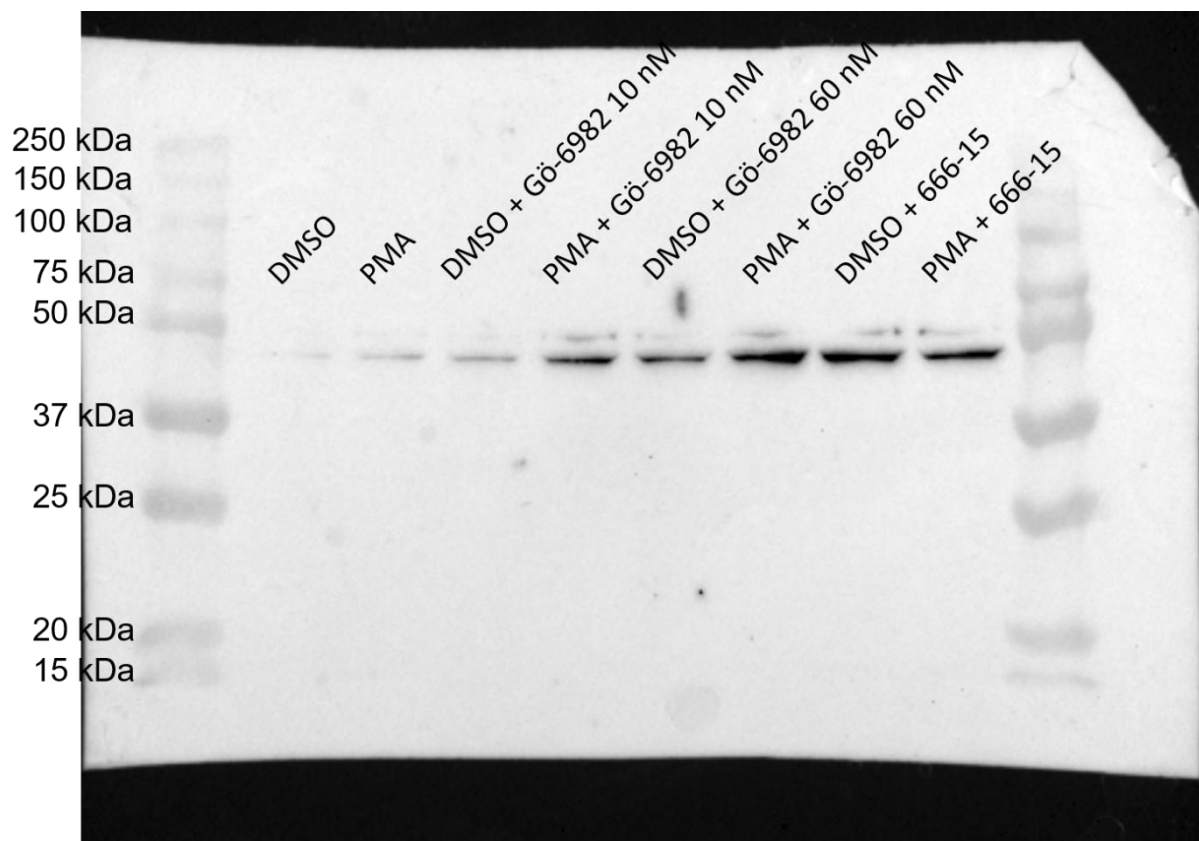

**Fig S3 (F)**

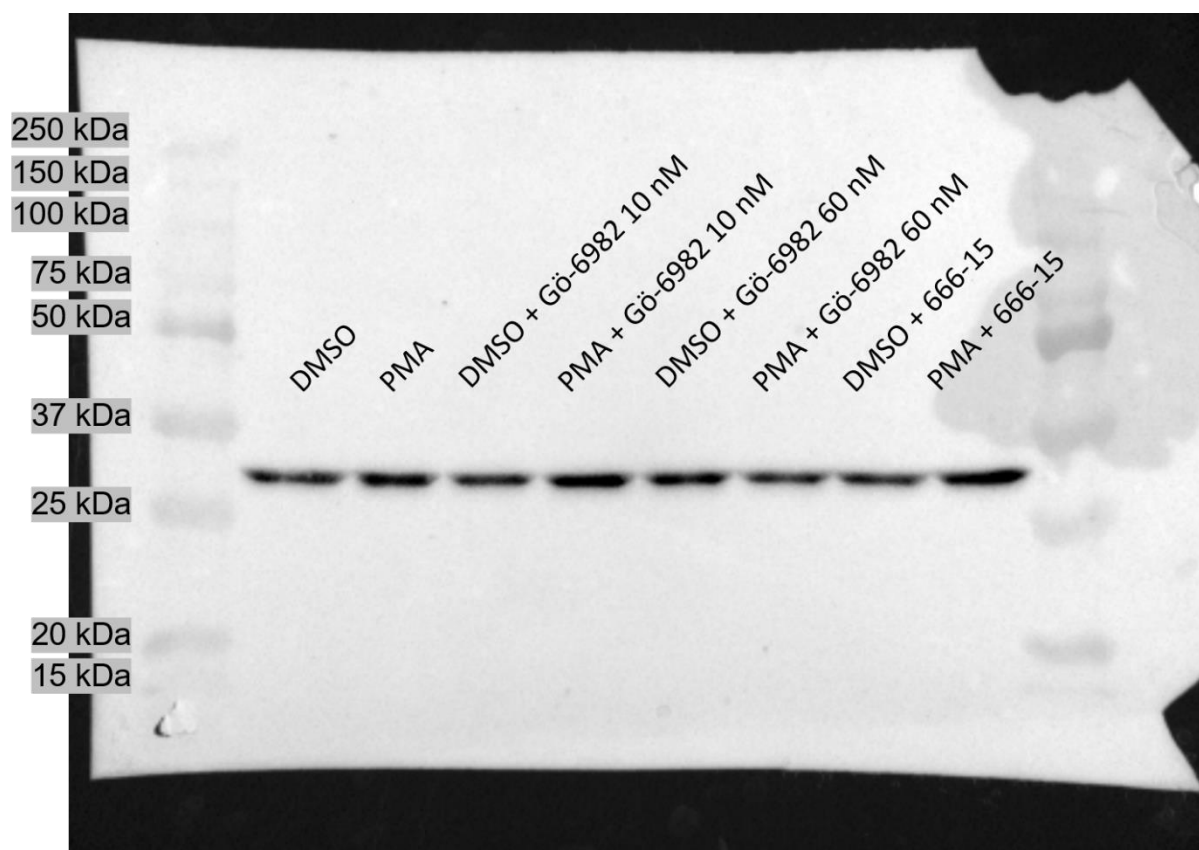

Supplement: S3 Fig — (A) Uncropped Western blot of GRK6 protein expression in Jurkat cells. The main GRK6 band was detected at approx. 70 kDa (molecular weight 66 kDa), a second band at less than 80 kDa. Stimulation with 100 nM PMA led to a distinct decrease in GRK6 protein expression after 6 h of stimulation. (B) Uncropped Western blot of actin β protein expression in Jurkat cells. After membrane stripping, actin β at approx. 40 kDa (molecular weight 43 kDa) was used as a reference protein. (C) Uncropped Western blot of GRK6 protein expression in Jurkat cells including stimulation with CREB inhibitor 666–15 (blot 1). Serum starved Jurkat cells were stimulated with 10 nM pan-PKC inhibitor Gö-6983, 60 nM pan-PKC inhibitor Gö-6983 and 100 nM CREB inhibitor 666–15 +/- 100 nM PMA as indicated for 6 hours. GRK6 was detected at approx. 70 kDa. (D) Uncropped Western blot of actin β protein expression in Jurkat cells including stimulation with CREB inhibitor 666–15 (blot 1). Serum starved Jurkat cells were stimulated with 10 nM pan-PKC inhibitor Gö-6983, 60 nM pan-PKC inhibitor Gö-6983 and 100 nM CREB inhibitor 666–15 +/- 100 nM PMA as indicated for 6 hours. Actin β was detected at approx. 40 kDa on a stripped membrane. (E) Uncropped Western blot of GRK6 protein expression in Jurkat cells including stimulation with CREB inhibitor 666–15 (blot 2). Serum starved Jurkat cells were stimulated with 10 nM pan-PKC inhibitor Gö-6983, 60 nM pan-PKC inhibitor Gö-6983 and 100 nM CREB inhibitor 666–15 +/- 100 nM PMA as indicated for 6 hours. GRK6 main band was detected at approx. 70 kDa. (F) Uncropped Western blot of actin β protein expression in Jurkat cells including stimulation with CREB inhibitor 666–15 (blot 2). Serum starved Jurkat cells were stimulated with 10 nM pan-PKC inhibitor Gö-6983, 60 nM pan-PKC inhibitor Gö-6983 and 100 nM CREB inhibitor 666–15 +/- 100 nM PMA as indicated for 6 hours. Actin β was detected at approx. 40 kDa on a stripped membrane. (PDF) [file pone.0247087.s003.pdf]
